# The Perceived Restorativeness of Differently Managed Forests and Its Association with Forest Qualities and Individual Variables: A Field Experiment

**DOI:** 10.3390/ijerph18020422

**Published:** 2021-01-07

**Authors:** Jenni Simkin, Ann Ojala, Liisa Tyrväinen

**Affiliations:** 1 Natural Resources Institute Finland, Latokartanonkaari 9, 00790 Helsinki, Finland; ann.ojala@luke.fi (A.O.); liisa.tyrvainen@luke.fi (L.T.); 2 Department of Forest Sciences, University of Helsinki, Latokartanonkaari 7, 000790 Helsinki, Finland

**Keywords:** forest management, biodiversity, forest qualities, individual variables, nature relatedness, psychological restoration, well-being, field experiment, perceived restorativeness

## Abstract

Despite increasing research knowledge about the positive well-being effects forests have on citizens, it is still unclear how the quality of forests and individual variables effect the well-being. This research investigated (1) the differences in restorative experiences (components being away, fascination, compatibility and extent, measured by perceived restorativeness (PRS)), and (2) how people evaluate forest qualities in four differently managed forests. Furthermore, this research studied (3) which individual variables (4) as well as forest qualities, explain the overall restorative experience (PRS-score from all components). Altogether, 66 volunteers were taken in small groups to each of the four forest sites once, after their day at work. The participants viewed the forests for 15 min and then walked inside the forests for 30 min. Their perceived restorativeness and perceptions about forest qualities were measured on-site after each visit. Most of the components of PRS differed between the three older forests compared to the young forest. The three older forests also had more preferred qualities, compared to the young commercial forest. From the individual variables, the nature relatedness positively explained the restorative experiences (PRS-score) in old-growth forest and in mature commercial forest. Beauty was the most important quality that explained PRS-score in all forests. Biodiversity positively explained the PRS-score, except in urban recreation forest. However, not all forest qualities need to be present in order to reach high perceived restorativeness and both a pristine or managed old forest can have high restorative values. Also, decaying wood does not seem to diminish forests’ restorative values, but there may be individual differences in its acceptance. Therefore, a greater attention to the overall versatility is needed when managing the forest used for outdooring.

## 1. Introduction

Nature is found by many studies to enhance well-being, restoration, vitality, as well as positive moods, decrease negative moods, and, eventually, reduce stress [1,2,3,4,5]. Even a short visit to a nature area is shown to have positive effects on stress recovery and a renewal of directed attention capacity compared to a visit in urban built areas [5,6]. Living in urban areas has been linked to an increased risk of mental health problems [7], whereas growing up near natural areas seems to decrease the risk of several psychiatric disorders, even those such as schizophrenia [8,9]. At the same time, the exposure to nature areas is becoming less frequent as up to 68% of the world’s population is expected to live in urban areas by 2050 [10]. Despite the research evidence of nature’s contribution to human well-being, social and environmental benefits of nature have often been subordinate to the economic objectives of urban planning [11,12,13].

In the European Union, forests cover 43% of land area, of which only 4% has not been modified by humans [14]. Finland is among the most forested countries in Europe, with about 77% of the land area being forests, of which about 86% is available for wood production [15]. However, the management for maximizing wood production generally lowers the quality of the landscape, at least for a certain time period [16]. Urban forests are usually managed from a recreational perspective, but in Finland, often rural and sometimes even in peri-urban areas, forest management intensity can be rather high because of timber production.

Impacts of forest management on landscape and recreation values have been extensively studied among outdooring people [16,17,18,19], but better understanding is needed on what qualities make the forest restorative, and how those qualities can be implemented in forest management. Therefore, this study explores to what extent people’s perceptions of restorativeness and preferred forest qualities coincide. More information is also needed regarding how the restorative qualities of different types of forests are perceived by different users. Once this connection is known, the information can be used when selecting forest management regimes.

## 2. Theoretical Framework

### 2.1. The Theory of Lansdscape Preferences and Restoration

There are several landscape theories used in preference research, such as the information model, to understand people’s perceptions and evaluations of different forest landscapes. These theories, however, do not focus directly on measuring the restorativeness of the nature, but instead explore and analyze different types of landscape values important for users [20]. There are, however, linkages between landscape theories and the most commonly used theory in environmental psychology studies, the Attention Restoration Theory (ART). Consistent to common landscape theories and ART is that the preferred and restorative landscape and environment needs to have certain types of characteristics. ART describes how nature environments influence human health and well-being through restoration. This ‘restoration’ refers to the processes of one recovering from something that has reduced one’s ability to cope with everyday tasks and demands [21]. According to the theory, there are two types of attention: voluntary, or directed attention, and involuntary attention [20]. Directed attention requires cognitive effort that can eventually lead to fatigue, while involuntary attention is effortless. Importantly, while in the involuntary mode, directed attention is able to rest [22]. According to ART, natural environments are especially suitable for restorative experiences. The restorative environment is identified to have four different components: being away, fascination, compatibility, and extent. Being away can be sensed when a place is different enough from one’s everyday environment, while fascination is effortless attention towards the environment. Compatibility means the sense that the environment supports one’s intended actions, and the component extent means that the environment needs to be rich and coherent enough [23,24,25]. To evaluate these four restorative components of an environment, the Perceived Restorativeness Scale (PRS) was developed [22,26].

### 2.2. Forest Preferences and Restorativeness of Forests

According to preference studies, people prefer forests with varying qualities. Forests that have large-dimensioned, tall trees and in which it is easy to walk, are preferred [27,28]. Furthermore, people appreciate mature forests more than young, good visibility, but still some undergrowth [16,29,30]. People also prefer lightly managed natural-looking forests or forests that they perceive as natural, and do not have clear signs of cuttings, such as logging residues or stumps [31,32]. People do not usually value dead or fallen trees, e.g., see References [17,18,33], and according to Frick et al. [34], the study by Stelzig [35] found that some people even had feelings of sadness about dead wood. Brighter forests are also more preferred than darker forests [32,36] and the feeling of safety is important [18,37]. In the study where forest stands around Europe were studied, the recreational value increased with stand age, and decreased with management intensity [38].

There are only a few experimental studies exploring how the management of forests affects people’s perceived restoration, with contradictory results. Takayama et al. [39] compared whether viewing dense or thinned forests has a different restorative effect. A thinned forest was described as more compatible, bright, and open compared to a dense forest, but Takayama et al. found no significant difference on restorative effects. Both forests were plantation forests and not pristine. Martens, Gutscher, and Bauer [40] studied the influence of tended and ‘wild’ forests on well-being. After a walk in these forests, the positive affect was greater and the negative affect smaller in a tended forest compared to a wild forest [40]. However, both forests were located in urban areas, and the ‘wild’ forest had also been out of commercial use for only six years. Furthermore, the increased stand density and shrubs—common in more natural forests—negatively influenced the perception of the psychological benefits of forest visits in a study by Tomao et al. [41], whereas Chiang et al. [42] found no difference on stress levels between different stand densities when observing forests from three-dimensional (3D) images. In studies by Carrus et al. [43] and Fuller et al. [44], the actual biodiversity—common in more natural forests—as well as the perceived biodiversity [45] in green areas, was found to be positively linked to psychological well-being. Overall, results on how people perceive naturalness are somewhat inconsistent. In a meta-analysis of studies conducted in lab and field conditions, contact with managed and wild nature had similar effects on emotional well-being [46], but a study involving watching photos and videos found that environments that were perceived more natural were also rated as more restoring [47]. Marselle et al. [45] also found that perceived restorativeness mediated the effects of perceived naturalness. Either way, people can value naturalness differently depending on the expectations towards the environments [48]. In addition, different cultural and geographical conditions, in which the concept of naturalness is used, may vary. Moreover, aesthetics has been seen to go hand in hand with naturalness [49], whereas beauty has been rated as one of the most important qualities in nature [50,51].

There are only a few studies that have looked at the connection between preferences and restorativeness and how forest management decisions affect restorativeness, and how forests are preferred or appreciated as a recreational environment. According to Laumann et al. [52], restorativeness predicted the preferences, but the study was conducted by watching videos. Han [53] found that restoration was not found to be an effective predictor for preferences or scenic beauty. Chiang et al. [42] found that people preferred the interior location of forest, which also provided the most optimal stress reduction out of edge or exterior. In a study by Martens et al. [40], appreciation did not differ between tended or wild forests even though the effect of well-being did.

### 2.3. The Effect of Individual Variables

Individual variables such as age, gender, education, childhood living environment, and relationship with nature may also have an effect on how people perceive nature. Several survey studies have been conducted to explore these associations. In a Swedish study by Jong et al. [54], the association of perceived green quality to self-reported well-being remained after adjusting the sociodemographic factors, while in a study by Ode et al. [55], women and older people saw greater aesthetic value and higher self-reported well-being in urban green spaces than men and younger people. Furthermore, Astell-Burt et al. [56] obtained results that only physically active middle-to-older aged adults received mental health benefits from green spaces. In addition, in a Finnish study, recreational and health benefits derived from green areas were more emphasized by women [57]. People also tend to prefer landscapes experienced during childhood [58], but Hinds and Sparks [59] did not find differences between people with a rural childhood living environment compared to urban in their connection with natural environments.

Associations of individual variables with perceptions of nature have been studied with only few field experiments. Tomao et al. [41] did not find a connection with gender, age, or level of education, to perceived restorativeness in urban forests. However, people with higher levels of nature connectedness were suggested to be positively connected to ‘awe-inspiring’ experiences in wild nature [60] or to have higher restorative effects in more natural environments (urban woodland over urban park [61]).

### 2.4. Objectives and Hypotheses

According to previous research evidence, forests are perceived as restorative. This study investigates (1) the differences in restorative experiences, and (2) how people evaluate forest qualities in four differently managed forests. Furthermore, it studies (3) which individual variables, (4) as well as forest qualities explain the restorative experience. The hypotheses are as follows:

1. The restorative nature experience is based on four components (fascination, being away, compatibility, and extent [20,26]). However, it is not known whether these components are perceived differently during actual visits to differently managed forest sites. It is hypothesized that there are differences on restorative experiences between the forest sites.
2. Several previous studies have identified a set of preferred forest qualities (e.g., beauty, species richness, naturalness, and brightness). However, there is a limited number of on-site studies with several forest sites, especially also including a pristine forest. It is hypothesized that these forest qualities differ in differently managed forests sites.
3. As previous research is limited regarding how peoples’ individual variables such as age, education, and nature relationship affect how they evaluate their restorative experience in differently managed forests, specific hypotheses are not formulated on this issue.
4. It is unknown if the preferred forest qualities explain the forest’s perceived restorativeness. This study hypothesized that the most important properties such us beauty, safety, brightness, biodiversity, and management significantly explain how restorative forests are perceived.

## 3. Materials and Methods

### 3.1. Study Sites and Their Selection

This study wished to select forests that are typically used for recreational purposes and represent forests in the region (Table 1). According to preference studies, a managed mature forest is often ranked high for recreation, therefore a mature commercial forest was chosen as one of the experimental forests. This study was also interested in how the natural state forest is perceived compared to a managed forest; therefore, an old-growth forest was chosen for this experiment. In the southernmost region in Finland (the Uusimaa region), the percentage of mature forests (81–120 years of age) is, however, only 15.4% and old-growth forests are scarce (more than 120 years of age, naturally developed for decades), making up only 3.5% of all forest areas. The majority of forests, 40.2%, are less than 40 years old and 40% are middle-aged (40–80 years of age) [62]. Therefore, a young commercial forest, aged around 40 years, was also chosen for this experiment.

To some extent, this study replicated the study design of Tyrväinen et al. [6], where the forest in Central Park, Helsinki, was found to be effective for stress reduction. Therefore, the same forest was included in this field experiment to serve as the control forest. To avoid the possible restorative effect of varying dominant tree species between the sites, only spruce-dominant forests (*Picea abies*) were included, similar to the control forest site in Helsinki Central Park.

There were also several other criteria, such as accessibility, size, and exclusion of other possible restorative elements such as water courses in the landscape [63,64] and varying terrain [65] when selecting the potential forests for the experiment. The detailed description of the study sites and selection criteria can be found in the article of Simkin et al. [66]. The chosen forests were all large forested areas of more than 100 hectares. The control forest was located in Helsinki and the other three in a rural area in the municipality of Sipoo located next to Helsinki.

**Figure 1:**
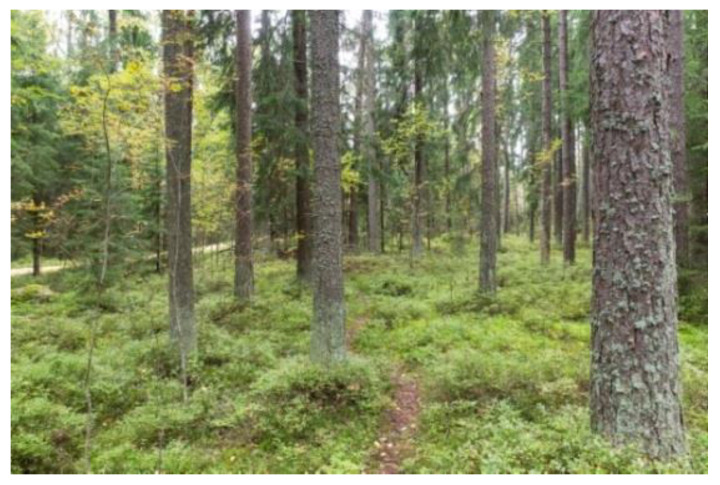
The urban recreation forest (Urban). This picture was published in Reference [66].

The control forest, Keskuspuisto (Central Park), is an urban recreation forest with high biodiversity values with a notable amount of dead wood (Figure 1). It has wide walking and cycling trails and a network of smaller footpaths across the area. 

**Figure 2:**
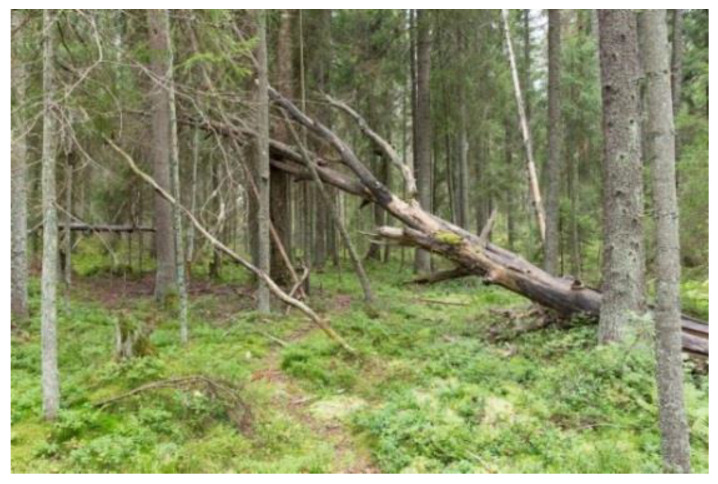
The old-growth forest (Pristine). This picture was published in Reference [66].

The old-growth forest was a large pristine protected forest area which has remained unmanaged for several decades (Figure 2). The forest is rich in biodiversity with an extensive amount of dead wood standing, and lying decaying trees, partly due to recent damage caused by the European spruce bark beetle (*lps typographus*). It also has multi-layered canopies and gaps.

**Figure 3:**
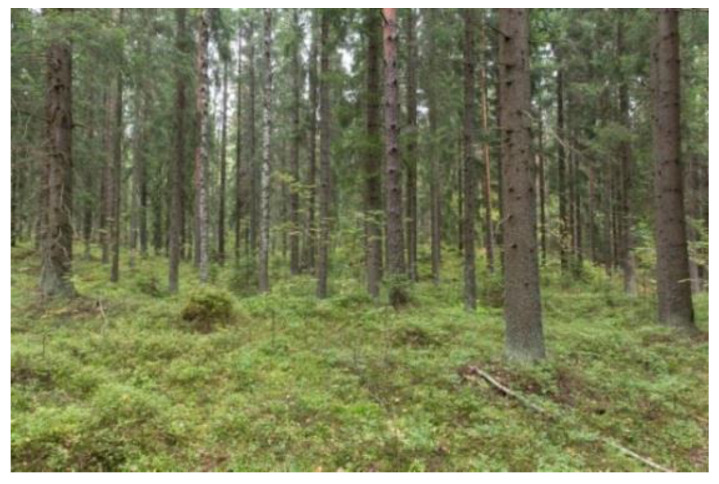
The mature commercial forest (Mature). This picture was published in Reference [66].

The mature commercial forest was located next to a recently harvested clear-cut area and its general appearance is more managed, with even-aged stand structure and less recreational infrastructure/trails than that of the urban recreation forest (Figure 3). Some dead wood has been left lying, which increases the biodiversity in the forest.

**Figure 4:**
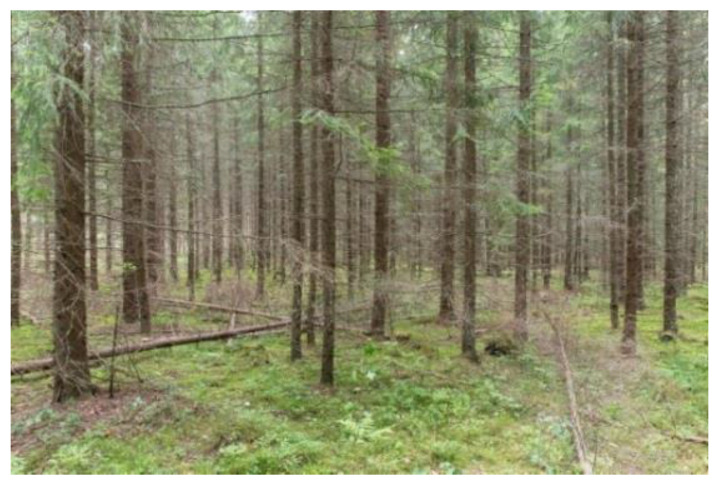
The young commercial forest (Young). This picture was published in Reference [66].

The young commercial forest was located near agricultural fields (Figure 4). The monoculture forest has been actively managed for timber production and thinning residues have been left on the site.

### 3.2. Recruitment

Volunteers were recruited, both women and men who had lived in the Helsinki metropolitan area for at least two years. Participants had to be full-time employees. Several selection criteria were used to ensure there were participants with varying backgrounds and interests in nature: age, gender, profession, background in nature conservation issues, studies related to nature, possible connections to the forest industry, and forest ownership. The final study sample consisted of 70 participants, 66 of whom visited all four study sites. Only those who visited all four sites were included in the final data analyses.

The participants were not informed that all the sites they would visit would be forests, but rather that the aim was to study what kind of nature is restorative. The participants signed written consent for their voluntary participation after receiving instructions on how the experiment would be performed and information on their rights based on principles of the Declaration of Helsinki, adopted by the World Medical Association. According to the Finnish Advisory Board on Research Integrity, an ethical review of the study was not required. The participants did not receive any incentives for participating in the study. A more detailed recruitment process was discussed in the article of Simkin et al. [66].

### 3.3. Experimental Procedure

All the forests were visited on random weekdays and in a random order. Each forest was coded to have a similar amount of first, second, third, and fourth visiting times in order to eliminate the order effect. A within-subject design was used where all participants visited each forest once in order to increase the validity of the study. One participant visited approximately one forest per week. However, as several participants needed to reorganize their scheduled study days due to their own timetable changes, some participants visited more than one forest during the same week. Nobody visited the forests in the same group (1–6 people) to avoid possible social effects with familiar people.

The participants were collected from the same meeting point in Pasila, Helsinki, and brought to the experiment sites by minivan each time. Prior to each visit, they knew only the city of the environment. The participants were asked to avoid discussions during the drive to the site. The trip to each site took 30–40 min as the length of each journey was controlled. Temporal changes on restoration during the experiment as well as perceived restorativeness after the experiment were measured with questionnaires in all forests. The questionnaires also included questions about how the participants experienced the surroundings. The results of the temporal changes are reported in Simkin et al. [66].

The participants were asked to be silent throughout the experiment and not to pick mushrooms, berries, etc. The experiment included 15 min of sitting in chairs on-site and a 30 min walk which was led by a researcher. Another researcher walked behind the group and carried equipment for noise measurement. The whole experiment took approximately three hours.

The experiments were conducted from August to October in 2016, and from April to June in 2017. A few visits were also made in September and October 2017. A more detailed experimental procedure is explained in Simkin et al. [66].

### 3.4. Measures

#### 3.4.1. Background Information

Gender, age, level of education, and average weekly working hours were asked (see Table S1 in the Supplementary Materials). Also, average health and physical condition were requested on a 5-point Likert scale (1 = very good; 5 = very poor).

#### 3.4.2. Relationship with Nature

Relationship with nature was requested using the questions: ‘How familiar are you with outdooring in forests?’ (on a 5-point Likert scale (1 = not at all familiar; 5 = very familiar)), ‘Is your work related to nature‘, and ‘Is your education related to nature?’ (with yes or no selections). The type of childhood living environment and whether the participants owned forests were also requested (see Table S1 in the Supplementary Materials). The participants also filled in the short version of the Nature Relatedness Scale (NR-6) [67]. The scale consisted of six items on a 5-point Likert scale (1 = strongly disagree; 5 = strongly agree). Items can be seen in Table S2 in the Supplementary Materials.

#### 3.4.3. Measures during the Experiment

##### Perceived Restorativeness Scale (PRS)

The Perceived Restorativeness Scale (PRS) was measured immediately after each forest visit. The PRS scale consists of 16 questions rated on a seven-point Likert scale (1 = not at all; 7 = totally) describing four components based on ART [26]. These components describe different aspects in restoration (see Table S3 in the Supplementary Materials for the scale items). As the study wished to explore how the differently managed forests differ, the mean scores from each component between the forests were compared. To discover how the different individual variables and forest qualities are associated with the overall perceived restorativeness, the mean score of all PRS-components (‘PRS-score’), used for example in a study by Hietanen et al. [68] and Marselle et al. [45], was calculated. To achieve this, reversed scores of the extent component were used.

##### Semantic Differential Method for Forest Qualities

A semantic differential method was used to study experienced qualities of differently managed forests. This method measures the subjective impressions of participants’ thoughts about each environment with opposing adjective pairs [69]. Twelve adjective pairs were asked to be rated immediately after each forest visit, of which eight were adapted from a study by Park et al. [1] into the following form: ‘Beautiful–Ugly’, ‘Safe–Scary’, ‘Natural–Artificial’, ‘Interesting–Dull’, ‘Calm–Restless’, ‘Harmonious–Chaotic’, ‘Pleasant–Unpleasant’, and ‘Bright–Dark’. In addition, three adjective pairs were added that were considered to be important according to previous study results: ‘Rich in biodiversity–Poor in biodiversity’ [43,45], ‘Managed–Unmanaged’ [39,40], and ‘Cheerful–Sad’ [34,35]. ‘Restorative–Stressful’ was also added so it could be compared with the PRS results.

##### Open Questions

To find out more precisely how the four forest sites met individual expectations or preferences, and what forest qualities participants experienced positively or negatively, after each forest visit, two open-ended questions were asked: ‘What did you like about this forest?’, and ‘What did you not like about this forest, or what disturbed you about this forest?’ The participants also had the possibility to express their own remarks freely at the end of the survey.

##### Other Measures

In order to find out whether the group situation affected participants, they were asked how alert they were of other people around on a scale of 1–7 (1 = not at all; 7 = completely). Using a similar scale, they were also asked how focused they were on the surrounding nature sounds in comparison to other sounds with a “Sound focus other than nature” test. In addition, the noise levels were measured during the experiment using the Larson Davis noise dosimeter, model 706RC (MTS Systems Corporation, Eden Prairie, MN, USA), and the temperature on-site, before and after each experiment (see Table S4 in the Supplementary Materials).

##### Statistical Analyses

Repeated measures of analysis of variance (ANOVA) was used to calculate the effects of the Perceived Restorativeness Scale (PRS) and semantic differentials after visits in different forests. The four within-subject factors were used: urban recreation forest (Urban), old-growth forest (Pristine), mature commercial forest (Mature), and young commercial forest (Young). For each PRS component and semantic differential, own models were performed to obtain all the contrasts with the reference categories.

As the original coded visiting order changed because several participants needed to reorganize their scheduled study days, we confirmed with measures of analysis of covariance (ANCOVA) that the visiting orders did not have an effect on the components of perceived restorativeness or the PRS-score. Paired samples t-test was used to calculate the differences on temperatures and sound focus other than nature and Kruskal–Wallis test was used to calculate the differences on average noise levels (dBA) between the forests.

To calculate how much the individual variables and forest qualities explained the overall perceived restorativeness in each forest, four different multiple regression models (one for each forest) were calculated. Only 13 variables could be included in the whole model due to the limited number of observations, as the risk for biased results increases if there are less than five observation units (66 participants) for each variable included in the analysis [70]. To simplify the interpretation of the results, first, the overall PRS-score for each forest from all PRS-components was calculated. In this analysis, reversed values of the extent component were used. The PRS-score was treated as a dependent variable in the regression models. The variables were added in three different steps to each model in hierarchical order. Gender, age, the level of education (dummy coded: 0 = ‘Other’ consisting of all education below the level of university of applied sciences degree, 1 = ‘Uni’ consisting of all education above the level of short-cycle tertiary education) and the type of childhood living environment (dummy coded: 0 = ‘countryside’ consisting also of sparsely populated areas, 1 = ‘City’ consisting of all other, more urban areas) were used as control variables. These variables were added in the first step. Temperature was also included in the model as it had correlations to the dependent variable in some forests. In the second step, variables that measure the relationship with nature were added: the short Nature Relatedness Scale (NR-6), familiarity of outdooring in the forest, and work related to nature. The variable ‘education related to nature’ was excluded as it correlated strongly with the variable ‘work related to nature’ (multicollinearity). The variable ‘forest ownership’ was also excluded from the model as it did not correlate with the dependent variable. For the third step, scores derived from adjective pairs describing forest qualities (semantic differentials): beauty, security, brightness, biodiversity richness, and managed, were added as they are commonly identified in the preference studies to describe forests that are preferred. For example, if the forest was rated ‘7’ on the pair ugly–beautiful, it was perceived as very beautiful.

In the final multiple regression model (step 3), the lowest tolerance value was 0.298, and the Variance Inflation Factor (VIF) value was 3.357, indicating acceptable multicollinearity with some concerns, and therefore the model is approximate (see a more detailed description in Table S5 in the Supplementary Materials). Each model obtained acceptable results, indicating no autocorrelation from the Durbin Watson test with values ranging between 1.698 and 2.630 (acceptable values 0–4).

Statistical analyses were conducted using the SPSS Statistics Version 25 (IBM, Corp., Armonk, NY, USA) and Excel (Microsoft, Redmond, WA, USA). The open questions were analyzed by using the Atlas.ti 8 Windows (Scientific Software Development GmbH, Berlin, Germany).

## 4. Results

### 4.1. Background Information

The participants were 26–65 years old (M = 43.38, standard deviation (SD) = 10.68), of which 39 were women, 27 men, and 74% had a higher education. They worked an average of 43 h per week (SD = 7.80), and the average self-evaluated health (1.77, SD = 0.80) and physical (2.32, SD = 0.88) condition were quite good.

### 4.2. Relationship with Nature

Most (67%) of the participants’ childhood living environment was in population centers. Twenty-one percent of the participants had received an education that was related to nature, 23% of participants worked in nature-related issues, and 9% were forest-owners. For 9% of participants, outdooring in forests was quite or somewhat unfamiliar, and it was quite or very familiar for 91%. The average score for Nature Relatedness Scale (NR-6) was 3.9 (SD = 0.61). Please see Table S1 in the Supplementary Materials for more detailed information.

### 4.3. Results from the Experiment

The mean sum scores were calculated for all four PRS components, overall PRS-score, and semantic differentials (Table 2), as well as for sound focus other than nature, temperature, and noise (Table S4 in the Supplementary Materials). The reverse scale items were taken into account. It is assumed that the group situation did not significantly affect the participants during the experiment, as a majority (84%) answered that they were not at all, very little, or fairly little focused on the other people around them. The reliability measured with Cronbach’s α for the PRS components are presented in Table 2 and discussed in more detail in Table S3 in the Supplementary Materials.

The correlations between the PRS, semantic differentials, sound focus other than nature, and temperature are presented in the Supplementary Materials, Tables S6–S9.

#### 4.3.1. Results of Repeated Measures ANOVA and Analysis of Covariance (ANCOVA)

When sphericity was violated according to Maulchly’s test, the estimates of sphericity were adjusted using the Greenhouse-Geisser correction.

##### Results of the Perceived Restorativeness Scale

**Figure 5:**
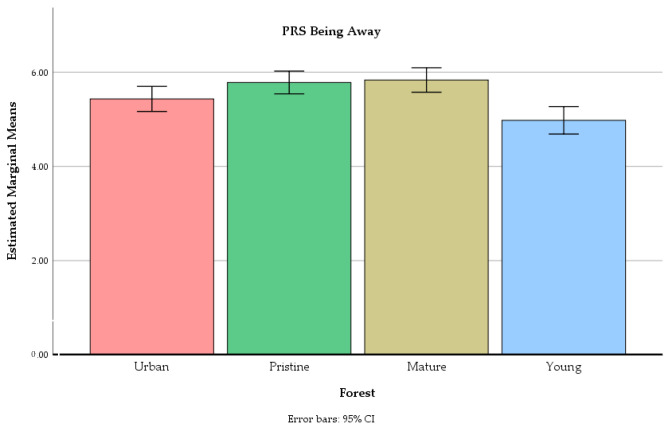
The mean values of PRS being away in four different forests at the end of the experiment. Error bars represent 95% confidence intervals.

**Figure 6:**
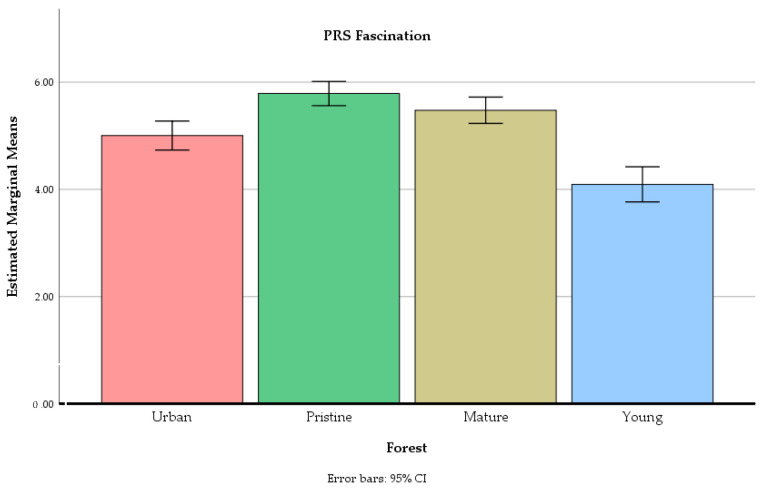
The mean values of PRS fascination in four different forests at the end of the experiment. Error bars represent 95% confidence intervals.

**Figure 7:**
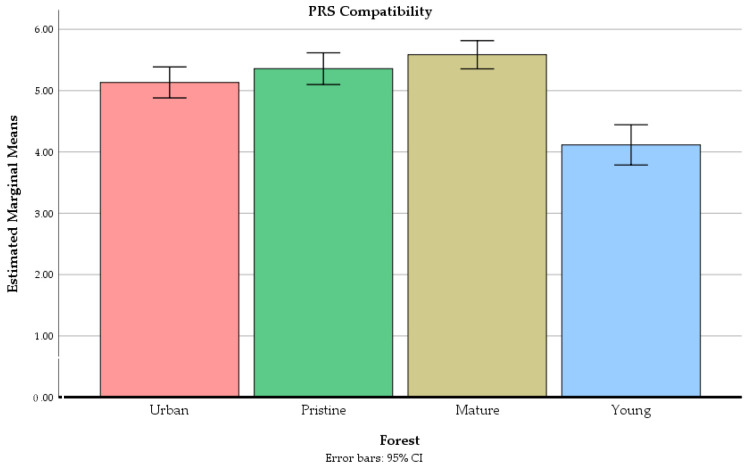
The mean values of PRS compatibility in four different forests at the end of the experiment. Error bars represent 95% confidence intervals.

**Figure 8:**
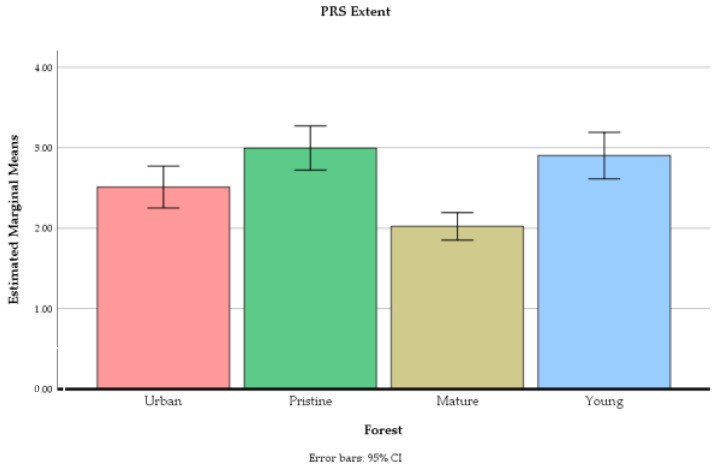
The mean values of PRS extent (not reversed) in four different forests at the end of the experiment. Error bars represent 95% confidence intervals.

The forest site had a significant main effect on ratings of the PRS being away F(2.65, 172.26) = 14.87, *p* < 0.01, the PRS fascination F(2.40, 156.09) = 46.02, *p* < 0.01, the PRS compatibility F(2.40, 156.00) = 33.12, *p* < 0.01, and the PRS extent F(2.51, 163.22) = 16.13, *p* < 0.01 (see Figure 5, Figure 6, Figure 7 and Figure 8).

Contrasts revealed several significant differences between the forests, from small to very large effect sizes (see Table 3). The being away feeling was stronger in all other older forests compared to the young commercial forest. It was also stronger in the mature commercial forest and in the old-growth forest, compared to the urban recreation forest.

The old-growth forest was perceived as more fascinating compared to all three other forests. The mature commercial forest was perceived as more fascinating compared to the young commercial forest, and to the urban recreation forest, which was again perceived as more fascinating compared to the young commercial forest.

Participants perceived the mature commercial forest as more compatible compared to the young commercial forest, and to the urban recreation forest. The old-growth forest and the urban recreation forest were perceived as more compatible compared to the young commercial forest.

Moreover, the feeling of extent was stronger in the old-growth forest and young commercial forest compared to the mature commercial forest. The urban recreation forest was perceived as more extent compared to the mature commercial forest but less extent compared to the old-growth forest and young commercial forest (see Table 3).

##### Results of Semantic Differential Adjective-Pairs

**Figure 9:**
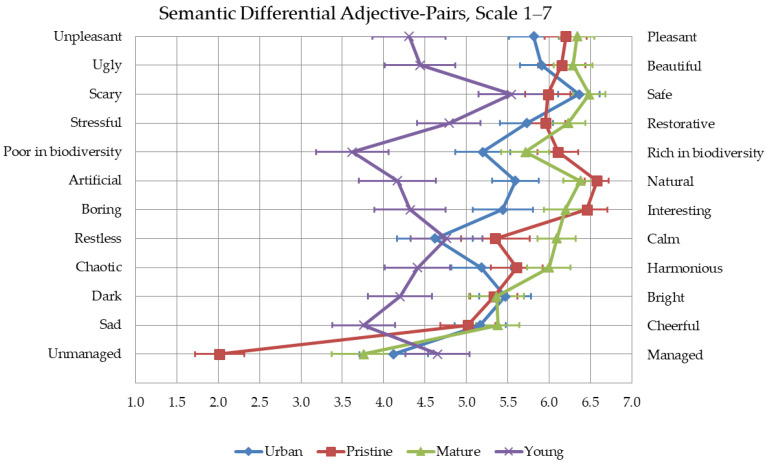
The mean values of adjective-pairs in four different forests. Error bars represent 95% confidence intervals.

The forest site had significant main effects on ratings of all semantic differential adjective-pairs, as follows: Pleasant–Unpleasant F(2.15, 137.90) = 39.34, *p* < 0.01, Beautiful–Ugly F(2.29, 148.87) = 38.22, *p* < 0.01, Safe–Scary F(2.25, 146.13) = 10.23, *p* < 0.01, Restorative–Stressful F(2.42, 260.09) = 19.35, *p* < 0.01, Rich in biodiversity–Poor in biodiversity F(2.65, 172.42) = 48.93, *p* < 0.01, Natural–Artificial F(1.91, 123.85) = 65.68, *p* < 0.01, Interesting–Dull F(2.49, 161.94) = 38.08, *p* < 0.01, Calm–Restless F(2.43, 158.20) = 13.80, *p* < 0.01, Harmonious–Chaotic F(2.54, 164.87) = 19.01, *p* < 0.01, Bright–Dark F(3.00, 195.00) = 17.48, *p* < 0.01, Cheerful–Sad F(2.64, 171.57) = 27.09, *p* < 0.01, and Managed–Unmanaged F(2.67, 173.82) = 46.44, *p* < 0.01 (Figure 9).

Contrasts revealed several significant differences between the forests, from small to very large effect sizes (Table 3). The mature commercial forest and the old-growth forest were rated more pleasant and interesting compared to the urban recreation forest and the young commercial forest, and the urban recreation forest more pleasant and interesting compared to the young commercial forest. The mature commercial forest was rated more beautiful compared to the urban recreation forest and the young commercial forest, whereas the old-growth forest and the urban recreation forest were rated more beautiful than the young commercial forest. The old-growth forest was rated the most unmanaged compared to all the other three forests, and the mature commercial forest more unmanaged compared to the young commercial forest.

The assessment of biodiversity differed significantly in all the forests, where the old-growth forest was assessed as having the highest biodiversity, followed by the mature commercial forest, then the urban recreation forest, and finally, the young commercial forest as having the least biodiversity. The young commercial forest was also rated less natural than all the other three forests, and the urban recreation forest was less natural compared to the old-growth forest and the mature commercial forest.

The mature commercial forest, the old-growth forest, and the urban recreation forest were rated brighter and more cheerful compared to the young commercial forest, while the mature commercial forest was rated more cheerful also compared to the old-growth forest. The mature commercial forest was rated calmer and more harmonious compared to all the other three forests. Moreover, the old-growth forest was rated calmer compared to the urban recreation forest and the young commercial forest, and more harmonious compared to the young commercial forest. The urban recreation forest was also rated more harmonious compared to the young commercial forest.

The mature commercial forest and the urban recreation forest were rated safer compared to the old-growth forest and the young commercial forest. The three older forests were evaluated more restorative compared to the young commercial forest, and the mature commercial forest was also rated more restorative compared to the urban recreation forest.

#### 4.3.2. Results from the Open Questions

In the urban recreation forest, mature commercial forest, and in the young commercial forest, the most frequently mentioned features that participants liked were the sounds of the forest and birds, and in the old-growth forest, the dead or decaying wood. Versatility, naturalness, brightness, species richness, and details were also frequently mentioned features. The number and proportions of remarks by participants for all the mentioned liked characteristics can be seen in Table 4.

Airplane noise was the most often mentioned issue that participants did not like in the old-growth forest, in the young commercial forest, and in the mature commercial forest. In the urban recreation forest, the most often mentioned issue the participants did not like was traffic noise.

The participants mentioned just a few other issues which they did not like in the old-growth forest and in the mature commercial forest (Table 5). However, in the urban recreation forest, 18% of the participants felt that there were too many other people and 14% of the participants felt that there were too many paths. In the young commercial forest, 32% of the participants stated that they did not like its features of a commercial forest. Many (26%) of the participants mentioned that the young commercial forest was too monotonous or dull, 20% of the participants did not like the dead or dried lower branches of the spruce trees, and 17% of the participants mentioned that they did not like the logging residues. Overall, 73% of the participants mentioned some qualities in the young commercial forest itself that irritated them, while the proportion of participants reporting negative features in the mature commercial forest was 17%, 18% in the old-growth forest, and only 15% in the urban recreation forest.

#### 4.3.3. Results of the Effect of Individual Variables and Forest Qualities on Restorativeness

The results for each variable in steps one and two can be seen in Table S5 in the Supplementary Materials. Step one was not significant in any of the forests. Step two was significant in the old-growth forest (*p* = 0.001) with a coefficient of determination of 27%, in the mature commercial forest (*p* = 0.012) with a coefficient of determination of 15%, and in the young commercial forest (*p* = 0.050) with a coefficient of determination of 14%. The overall model (step three) was very significant (*p* < 0.001) in all four forests. The corresponding coefficient of determination was 68% in the old-growth forest, 52% in the mature commercial forest, 33% in the urban recreational forest, and 63% in the young commercial forest of the variation of perceived restorativeness (Table 6).

According to the final model (step three), in the old-growth forest, nature relatedness had a positive association, but the higher level of education had a negative association with perceived restorativeness. There was also a positive association with nature relatedness to perceived restorativeness in the mature commercial forest, but also the higher temperature was positively associated with perceived restorativeness.

The most important forest quality increasing perceived restorativeness in all the forests was perceived beauty. The association was positive in all four forests (see Table 6). In the old-growth forest, the quality ‘rich in biodiversity’ was also positively connected to perceived restorativeness as well as the quality of ‘managed’. The quality of ‘rich in biodiversity’ had a positive association with perceived restorativeness also in the mature commercial forest and in the young commercial forest.

There was no evidence of associations between gender, age, or familiarity of outdooring in the forest and perceived restorativeness according to the model. Correlations or nonparametric test values of the null hypothesis between the PRS-score and temperature, individual variables, and five adjective-pairs that were chosen in the multiple regression model are presented in the Supplement Materials, Tables S10–S13.

## 5. Discussion

Forest management affects experiential qualities of forests, but still only a few studies have compared restorativeness of different kinds of forests or have explored the link between restorativeness and preferences for different forests. This field experiment investigated how people perceive four differently managed forests and what the linkages of individual variables and the forests qualities to restorativeness are.

### 5.1. The Four Components of the PRS Are Perceived Differently in All Forests

The results confirm the first hypothesis of this study, that the components of the PRS are perceived differently in four differently managed forests. Three of the PRS-components (being away, compatibility, and fascination) were perceived higher, with a medium to a very large effect size in all three older forests compared to the young commercial forest. This result is in line with the findings of preference studies that older forests are more preferred than young forests, e.g., References [17,30]. The three older forests in this experiment all had generally preferred forest qualities, such as old and large trees, a natural state or appearance, and no clear signs of forest management [16,17,31]. All of these forests also had good visibility and not too much undergrowth.

The old-growth forest was perceived as very compatible, fascinating, and the feeling of being away was strong, but it was also experienced more extent than the other two older forests. In fact, the old-growth forest was perceived equally extent as the young commercial forest. Although the average value (3 = quite little extent) of extent was not very high in the old-growth forest, it suggests that people have more mixed views about the old growth forest. They may find it confusing, having distractive features or may experience it to be even slightly chaotic. However, this is not surprising due to the extensive amount of standing and lying dead wood in the forest. A large amount of dead wood is not often found in managed forests in Finland that are the most familiar recreation environments for Finns. The answers from the open questions also support the findings on extent, as several participants commented that the old-growth forest looked very diverse and interesting, but also rough and even confusing. Some wondered why there was so much dead wood, mentioned they did not like it, and one felt sad about it. However, there were more people who preferred the dead wood. Previous studies have also reported divided opinions regarding dead wood [34,72]. Today, the increased awareness of the ecological importance of dead wood may have also improved its acceptance [18,33,73].

Some differences were also found on perceived restorativeness between the old-growth forest and the mature commercial forest, both of which were in rural locations. The evaluations of the PRS components showed that mature commercial forest was perceived less extent than the old-growth forest, but also less fascinating. According to ART, the environment needs to meet all four components of the PRS in order to be restorative. The results of this study indicate, however, that if one component is lacking, another component can compensate for it. In other words, even though the old-growth forest might have been ‘too’ extent, at the same time, it was experienced as so fascinating that it did not matter. Correspondingly, the mature commercial forest was not perceived as fascinating as the old-growth forest, but as it was experienced as coherent, it probably compensated for the lack of fascination. This result is supported by the previous study results from these same forests where the temporal change in restoration was equal in both of these forests [66]. Moreover, the open answers also supported the results: in the old-growth forest, its naturalness, decaying wood, and details were especially appreciated, while in the mature commercial forest, the brightness and ease of walking were appreciated. In both forests, species richness and diversity emerged as positive responses. Nevertheless, it is important to note that flight routes ran more often over both the young commercial forest and the old-growth forest than the other two forests, which might have also affected the extent results in these forests. Participants mentioned in the open questions that the airborne noise irritated them, but as the amount of airborne noise was not specifically measured, it is not known whether this affected the overall restorative experience.

There were also some differences between the urban recreation forest and the other two older forests. The mature commercial forest and the old-growth forest were rated higher in the PRS components fascination compared to the urban recreation forest. The urban recreation forest was located close to the city center, and therefore may have been familiar to many of the respondents and be less fascinating. This conclusion is supported by the result of the being away component, which was significantly lower in the urban recreation forest compared to other older forests. Additionally, the compatibility component was higher in the mature commercial forest compared to the urban recreation forest. All these results are probably linked to the location of the study areas, as forests outside cities in rural areas are suggested to provide more restorative effects than urban forests [57,64,74]. Urban recreation forests typically have many other outdooring people [75], more traffic noise [76], and a denser trail network than rural forests. These issues were mentioned as negative in the urban recreation forest by several participants in the open questions. As previously mentioned, only 15% of participants found something negative about the urban recreation forest itself, which indicates that the typical characteristics (paths, outdooring people, noise, etc.) for urban forests caused the lower result in perceived restorativeness, compared to almost similar aged forests in rural locations.

### 5.2. The Preferred Forest Qualities Were Identified More in Older Forests

The results also confirm the second hypothesis that the preferred forest qualities recognized in previous preference studies differed between differently managed forests. The young commercial forest ranked the lowest by many qualities. It was typically managed, even-aged forest, with a little variation. It was not perceived very interesting, pleasant, harmonious, or rich in biodiversity according to the adjective-pairs. This result can be reflected in the Kaplan and Kaplan Information model [20], where the elements mystery, coherence, legibility, and complexity must be met in order for the environment to be appreciated. With the lack of these elements, there was not enough potential to sustain interest and urge participants to explore the forest further. According to the adjective-pairs, the young commercial forest was perceived as less natural, beautiful, and bright than other forests, and it was experienced as somewhat sad. In the open questions, participants expressed that, for example, the forest looked dull and like a typical commercial forest. These results describing the young commercial forest were consistent with several preference studies, e.g., References [17,29,31].

Only two adjective-pairs were rated similarly in the young and older forests. The young commercial forest was perceived as restless as the urban recreation forest and as safe as the old-growth forest. The urban location with more visitors and traffic noise in the urban recreation forest, and the logging residues lying around in the young commercial forest probably explain this restlessness. Whereas the extensive amount of lying and standing dead wood in the old-growth forest might have affected the feeling of safety and thus, explain its lower score of safety [18,37]. However, the explanation as to why the young commercial forest was also perceived less safe might be more complex. Perhaps the logging residues lying around made the general appearance seem restless and unsafe and also difficult to move if one was in danger. This suggestion is in line with the results of Hertzog and Kutzil [77], where the feelings of entrapment could mediate danger, and that visual access correlates to fear. Moreover, Appleton’s [78] theory of prospect-refuge can also explain why the young commercial forest was perceived less safe and not so preferred, as it was dark in places and there was no variation in open and closed places where one can hide but still be able to observe the surroundings.

Some differences as to how the qualities were perceived were also found between the three old forests. Despite quite different qualities valued between the old-growth and mature commercial forests, they were both perceived as restorative according to the adjective-pair ‘restorative–stressful’. They were also perceived as beautiful, pleasant, bright, interesting, and natural—of which the last is an interesting result as the mature commercial forest was less natural than the old-growth forest. Tyrväinen et al. [57] obtained results with naturalness being one of the most important features associated with favorite places. However, the fact that the mature commercial forest had some traces of forest management seems not to have affected the experienced naturalness. The mature commercial forest was somewhat older than recommended in the forest management guidelines for regeneration in southern Finland [79], and therefore it probably had more coarse woody debris and dead wood than on average. However, the mature commercial forest in this study was perceived as more natural than the urban recreation forest even though the urban recreation forest had a bit more undergrowth and other tree species than the mature commercial forest. These results indicate that the naturalness was perceived as a larger concept than only linked to forest management and includes also recreational infrastructure, existence of other visitors, and soundscape. Furthermore, results from the semantic adjective-pairs showed that the urban recreation forest was perceived as less restorative than the mature commercial forest but as restorative as the old-growth forest, even though it received lower values than the old-growth forest from the evaluations of the PRS components fascination and being away. The adjective-pair ‘restorative–stressful’ is not able to measure perceived restorativeness as detailed as the PRS, but it seems to be indicative. According to the adjective-pair ‘safe–scary’, the urban recreation forest was perceived safer than the old-growth forest, which probably increased its perceptions as restorative forest. Hence, this study suggests that the feeling of safety also affects the perceptions of restorativeness in the forest and is therefore an important quality. As the urban forest was located close to residential areas and there were lots of paths, there was no danger of one being lost. Still, despite there being other outdooring people, one could feel safe inside the forest without being noticed by others, which again supports the theory of prospect-refuge [78].

### 5.3. Some Individual Variables Affect Overall Perceived Restorativeness (PRS-Score) in Differently Managed Forests

As previous research was limited regarding how people’s individual variables affect how they restore in differently managed forests, this study did not formulate specific hypotheses on this issue but explored the possible associations with a hierarchical regression analysis. Participants evaluated the sites rather similarly despite their age, gender, and childhood living environment, as these variables did not affect perceived restorativeness in differently managed forests. However, in the final model, a negative association was found between highly educated people (n = 49) and perceived restoration in the old-growth forest. There was no larger variation (SD) in the mean value of all the participants’ PRS-scores in the old-growth forest compared to other forests and the test of homogeneity of variances revealed that there was a difference in variation between the two educational level groups in the old-growth forest but also in the mature commercial forest. Therefore, this study could not find the reason for this result and needs further investigation. Nevertheless, the participants with a higher educational level answered lower to almost all of the questions compared to those participants with a lower educational level, which is in line with previous study results [80]. However, the result that education would have an effect to the results cannot be generalized as there were very few participants that had not completed at least the high school level.

This study also explored whether a participant’s relationship with nature (nature relatedness, familiarity of outdooring in a forest, and work related to nature) explained perceived restorativeness in differently managed forests. The study did not find an association between the familiarity of outdooring in a forest, or work related to nature, and perceived restorativeness, but for nature relatedness, quite a strong positive association with perceived restorativeness was found in the old-growth forest. This result is in line with a previous study by Davis and Gatersleben [60], where people with higher levels of nature connectedness were suggested to be positively connected to ‘awe- experience’ in wild nature. However, there was also a similar association between nature relatedness and perceived restorativeness in the mature commercial forest. This indicates that the mature commercial forest in this study also fulfils the needs for the restoration of people with strong nature relatedness. This result is in line with the preference studies, according to which people prefer both the natural and natural-looking forests. Finally, whether people with a stronger nature relationship need a more natural environment in order to be restored, or whether these people are able to restore more in a natural forest than people with a weaker nature relationship, remains unclear and should be studied further.

The standard deviations for the PRS components, PRS-score, and all adjective-pairs, except for calm–restless and managed–unmanaged, were largest in the young commercial forest, indicating that this forest divided opinions the most.

### 5.4. The Qualities Are Important in Explaining Restorativeness

The expectations in hypothesis four, that the different forest qualities: beauty, safety, brightness, biodiversity, and management, derived from the adjective-pairs would explain the overall perceived restorativeness (PRS-score), was partly supported. From these adjectives, beauty explained most of perceived restorativeness in all four forests. Beauty was also the only quality from the five qualities that significantly explained restorativeness in the urban recreation forest. This result is interesting, as Han [63] found that restoration was not an effective predictor for preferences or scenic beauty. Moreover, richness in biodiversity explained the restorativeness in the three other forests, except in the urban recreation forest, and, interestingly, more in the young commercial forest compared to the mature commercial forest and the old-growth forest, even though the forest was not rated very rich in biodiversity. However, as there was some biodiversity, such us flowers, lichen, and mushrooms, and as the forest was otherwise perceived quite dull, it seems that the participants drew attention to the biodiversity, in which case it gained more weight in the model and appeared to affect perceived restorativeness. The strong connection of perceived biodiversity with perceived restorativeness is interesting against the quite mixed previous research results on perceived naturalness and biodiversity, though they are in line with the study results of Marselle et al. [45] and Carrus et al. [47]. Perhaps the biodiversity can be difficult to experience through pictures or videos that have been commonly used in conducting preference studies. It may be that a person’s holistic experience of being in nature is different in real nature than in virtual nature [81].

Brightness did not explain restorativeness in any of the forests and not even in the young commercial forest, despite that the thickness of the forest made it quite dark, which is suggested to be one reason why the spruce forest is generally less preferred than the pine forest [32]. Safety did not explain the restorativeness in any of the forests either, even though it is known to be an important quality. However, perhaps the participants were able to focus on other issues as the experiment was led by the researcher and they were not alone in the forests. In addition, this study found that the forest management was associated with restorativeness in the old-growth forest. The more the participants felt this forest was managed, the more they felt restored. As the forest was quite unmanaged, this result means that for some participants, it was too unmanaged. There were lots of fallen trees and some participants felt the forest was already too extent, which was probably seen in the higher score of the extent component.

## 6. Strengths, Limitations, and Conclusions

### 6.1. Strengths and Limitations

One of the strengths of this study is its relatively large sample size (n = 66) compared to previous experiments conducted in this field [82,83], and the fact that the participants were both women and men, e.g., References [3,6,82,83,84]. Moreover, the experiment was conducted in real nature with full-time employees who arrived at the experiment after their working day. Sample representativeness was satisfactory with regard to the age structure in our experiment in comparison to the working age population in the municipality of Helsinki. There were, however, more women and participants with a higher education in the sample of this study according to the Official Statistics of Finland [85], and therefore one should be careful with generalizing the results. The study might also have had participants more interested in nature, and therefore with a stronger relationship with nature than average.

There were also some limitations when selecting the actual sites, though the experiment itself was successful. Although only spruce-dominated forests were deliberately included, it is also a limitation because of the no variation in the main tree species. There was also a rather limited variation in the management options as the experiment excluded, for example, regeneration cuttings. It was also difficult to find forests in or near the Helsinki Metropolitan area that would be relatively easy to access and fulfil the selection criteria for this study. Moreover, there was also more airborne noise in the study sites which could not be predicted in advance.

### 6.2. Conclusions

The results of this study indicate that forest management has an effect on perceived restorativeness and preferences, and that the outcome might also depend on people’s individual differences.

The results show that the restorative forest consists of different qualities and not all qualities have to be met in order for the forest to be restorative. One forest can be more fascinating, compatible, offer a more effective escape from routines, or even be more extent than the other, and still have a similar restorative effect on people. As previous preference studies indicate that the average outdoor recreationist mostly appreciates qualities common to a lightly managed forest [16,32], and that the restorative qualities of the forest may be improved with some forest management [39,40], the results of this study add that an untouched pristine old-growth forest can also be as restorative as a managed mature commercial forest.

According to the results of this study, the young commercial forest is not the best forest for restoration purposes nor is it a preferred environment for outdoor recreation. People prefer larger and older trees and a forest with more complexity. In this perspective, the rotation cycles in nearby urban and tourism areas in Finland should be extended as the forests are now often already regenerated between the ages of 60 and 80, before they provide well-being affects most effectively.

The perceived species richness significantly explained the perceived restorativeness in old-growth, mature commercial, and even in young commercial forests. This may indicate that biodiversity is an important variable in order to create a restorative forest, and even though a forest is not perceived as very fascinating and compatible, as the young forest was not, the biodiversity draws ones attention and helps involuntary attention to enter (ART). Moreover, as the beauty explained the perceived restorativeness in all forests, it clearly indicates its meaningfulness. Unlike in previous survey studies [17,18,33], the dead and decaying wood did not diminish the perceived appreciation and thus the perceived beauty of the old-growth forest. As landscape preference and scenic beauty assessments are traditionally based on visual input from the environment, those results in real nature, where other senses also contribute to evaluations, can differ from the visual preference evaluations. When, for example, seeing the dead wood in pictures, one cannot observe the biodiversity so closely as in real nature, nor see the movement and hear the sound of birds and other animals.

It also seems that the mature commercial forest is sufficient to fulfil the restoration needs of strongly nature-related people. This is a positive result in a nation where forestry is practiced in a large part of the country. However, as Ahtikoski et al. [86] state, it is possible to combine both forestry and outdoor recreation in the same area but required management changes typically cause reduction in timber production values.

### 6.3. Implementation Guidelines and Future Recommendations

The results of this study provide new information for land use planning and the management of recreational forests. They also strengthen those earlier results that individual differences and needs should be recognized in city planning and forest management.

As most of the participants stated that they were irritated about too many other people being present, or trails in the urban recreation forest, this study concludes that it is important that there are enough recreation forests so that the pressure of recreation does not become too high and that the trail network is wide enough but not too dense.

In future, it would be important to study whether different groups of people, for example, stressed or depressed individuals, need different types of forests in order to restore themselves, or whether they value different qualities. It would also be important to study the long-term effects of forest visits and also explore how nature relatedness changes over time among people in urban and rural areas.

## Figures and Tables

**Table 1:** Forest characteristics. Explanation of tree species: Norway spruce (*Picea abies*), Scots pine (*Pinus sylvestris*), European white birch and Downy birch (*Betula pendula* and *Betula pubescens*), Common aspen (*Populus tremula*), and European rowan (*Sorbus aucuparia*).

Forest Site	Urban	Pristine	Mature	Young
Location	Helsinki/Urban	Sipoo/Rural	Sipoo/Rural	Sipoo/Rural
Average age (years)	95	>120	100	40
Tree height (m)	26	33	27	16
Diameter breast height (cm)	30	35	28	16
Stand volume (m^3^/ha)	370	524	403	299
Dominant tree species	Norway spruce	Norway spruce	Norway spruce	Norway spruce
Other tree species	pine, birch, aspen, rowan	pine, birch, aspen, rowan	pine, birch, aspen, rowan	pine, birch, rowan

**Table 2:** Scale statistics of the four components in PRS, PRS-score and semantic differentials (adjective-pairs).

Forest Site	Urban			Pristine			Mature			Young		
Measures	Mean	SD	α	Mean	SD	α	Mean	SD	α	Mean	SD	α
*After walking*												
PRS being away	5.43	1.08	0.52	5.78	0.98	0.61	5.83	1.05	0.65	4.98	1.18	0.71
PRS fascination	5.00	1.10	0.90	5.79	0.92	0.89	5.48	1.00	0.89	4.09	1.33	0.92
PRS compatibility	5.13	1.03	0.90	5.36	1.05	0.90	5.58	0.94	0.91	4.12	1.34	0.93
PRS extent (not reversed)	2.51	1.06	0.74	3.00	1.12	0.63	2.02	0.70	0.47	2.90	1.18	0.75
PRS-score (extent reversed)	5.21	0.88	0.93	5.46	0.82	0.90	5.68	0.75	0.91	4.46	1.09	0.94
(7) Pleasant–Unpleasant (1)	5.79	1.22	-	6.20	1.03	-	6.33	0.85	-	4.35	1.81	-
(7) Beautiful–Ugly (1)	5.91	1.06	-	6.15	1.15	-	6.29	0.96	-	4.44	1.72	-
(7) Safe–Scary (1)	6.36	1.02	-	5.98	1.10	-	6.48	0.79	-	5.55	1.63	-
(7) Restorative–Stressful (1)	5.73	1.30	-	5.95	0.98	-	6.23	0.86	-	4.79	1.56	-
(7) Rich–Poor in biodiversity (1)	5.20	1.35	-	6.11	1.01	-	5.71	1.17	-	3.62	1.77	-
(7) Natural–Artificial (1)	5.59	1.15	-	6.58	0.58	-	6.38	0.84	-	4.17	1.89	-
(7) Interesting–Dull (1)	5.44	1.49	-	6.45	1.01	-	6.20	1.07	-	4.32	1.75	-
(7) Calm–Restless (1)	4.62	1.85	-	5.35	1.69	-	6.09	0.94	-	4.76	1.77	-
(7) Harmonious–Chaotic (1)	5.18	1.47	-	5.61	1.28	-	6.00	1.07	-	4.41	1.60	-
(7) Bright–Dark (1)	5.47	1.27	-	5.33	1.17	-	5.36	1.35	-	4.20	1.57	-
(7) Cheerful–Sad (1)	5.17	1.27	-	5.02	1.35	-	5.38	1.06	-	3.76	1.54	-
(7) Managed–Unmanaged (1)	4.12	1.70	-	2.02	1.21	-	3.76	1.58	-	4.65	1.56	-

Note: α = Cronbach’s alpha. SD = Standard Deviation.

**Table 3:** Results of simple contrasts in repeated-measures analysis of variance (ANOVA), F statistics.

Measure	Forest Site	Urban vs. Pristine	Urban vs. Mature	Urban vs. Young	Pristine vs. Mature	Pristine vs. Young	Mature vs. Young
PRS being away	F	5.63 *	7.31 **	7.27 **	.25	34.39 **	30.86 **
	r ^1^	0.28	0.32	0.32	0.06	0.59	0.57
PRS fascination	F	31.58 **	13.40 **	25.19 **	8.62 **	91.74 **	62.37 **
	r ^1^	0.57	0.41	0.53	0.34	0.77	0.70
PRS compatibility	F	2.43	13.25 **	30.37 **	3.54	48.06 **	60.98 **
	r ^1^	0.19	0.41	0.56	0.23	0.65	0.70
PRS Extent (not reversed)	F	8.65 **	15.48 **	4.17 *	56.06**	0.36	30.56 **
	r ^1^	0.34	0.44	0.25	0.68	0.07	0.57
Pleasant–Unpleasant	F	4.01 *	9.97 **	31.08 **	1.21	59.05 **	80.95 **
	r ^1^	0.24	0.37	0.57	0.14	0.69	0.75
Beautiful–Ugly	F	2.06	7.37 **	39.19 **	0.82	52.94 **	72.39 **
	r ^1^	0.18	0.32	0.61	0.11	0.67	0.73
Safe–Scary	F	4.98 *	0.71	13.31 **	16.63 **	3.83	19.54 **
	r ^1^	0.27	0.10	0.41	0.45	0.24	0.48
Restorative–Stressful	F	1.26	10.67 **	14.80 **	3.36	25.26 **	48.77 **
	r ^1^	0.14	0.38	0.43	0.22	0.53	0.65
Rich in biodiversity–Poor in biodiversity	F	18.33 **	7.99 **	39.73 **	4.70 *	99.40 **	78.33 **
	r ^1^	0.47	0.33	0.62	0.26	0.78	0.74
Natural–Artificial	F	45.73 **	36.46 **	36.65 **	2.95	107.34 **	87.10 **
	r ^1^	0.64	0.60	0.60	0.21	0.79	0.76
Interesting–Dull	F	22.22 **	15.77 **	17.12 **	2.40	74.23 **	80.11 **
	r ^1^	0.50	0.44	0.46	0.19	0.73	0.74
Calm–Restless	F	5.41 *	40.56 **	0.21	13.24 **	5.24 *	42.21 **
	r ^1^	0.28	0.62	0.06	0.41	0.27	0.63
Harmonious–Chaotic	F	3.98	16.91 **	8.78 **	6.42 *	22.90 **	47.63 **
	r ^1^	0.24	0.45	0.35	0.30	0.51	0.65
Bright–Dark	F	0.58	0.39	32.31 **	0.02	25.21 **	30.54 **
	r ^1^	0.09	0.08	0.58	0.02	0.53	0.57
Cheerful–Sad	F	0.55	2.07	43.92 **	4.07 *	29.17 **	64.24 **
	r ^1^	0.09	0.18	0.63	0.24	0.56	0.71
Managed–Unmanaged	F	105.56 **	2.64	3.54	59.58 **	138.51 **	12.50 **
	r ^1^	0.79	0.20	0.23	0.69	0.82	0.40

Note. ** F is significant at *p* < 0.01 level. * F is significant at a level of *p* < 0.05. ^1^ = r is the effect size, the relationship between the independent and dependent variables, ranging from 0.00 to 1.00. The interpretation of effect sizes is as follows: small > 0.10, medium > 0.30, large > 0.50, and very large > 0.70 [71].

**Table 4:** The number and proportion of characteristics mentioned that people liked in the forests. The stronger green, the more liked responses, and the stronger red, the less liked responses.

	Urban		Pristine		Mature		Young	
	no.	%	no.	%	no.	%	no.	%
Dead or decaying wood	7	11	24	36	3	5	1	2
Natural	9	14	23	35	3	5	1	2
Sounds of the forest and birds	18	27	18	27	19	29	19	29
Versatile	14	21	18	27	13	20	2	3
Species richness	7	11	17	26	13	20	2	3
Bright	11	17	9	14	16	24	14	21
Fascinating/awe/details	8	12	16	24	9	14	2	3
Old/large trees	5	8	8	12	13	20	2	3
Oldness	4	6	12	18	6	9	0	0
Serene/peacefulness	7	11	9	14	8	12	7	11
Beautiful	6	9	8	12	9	14	5	8
Easy to walk	8	12	3	5	9	14	7	11
Prospect/openness	3	5	4	6	7	11	2	3
Accessibility	7	11	0	0	1	2	1	2
Scents	1	2	4	6	6	9	4	6
Unmanaged	1	2	6	9	1	2	1	2
Different ages of trees	3	5	5	8	3	5	1	2
Compatibility	2	3	1	2	1	2	0	0
Extent	0	0	2	3	0	0	0	0
Space	2	3	0	0	0	0	0	0
Orderliness	0	0	0	0	0	0	2	3
Safe	1	2	0	0	1	2	0	0
Managed	0	0	0	0	0	0	1	2
Culture	0	0	0	0	0	0	1	2
Refuge/hide	0	0	1	2	0	0	0	0
No answer	1	2	0	0	0	0	0	0

**Table 5:** The number and proportion of characteristics mentioned that people did not like in the forests. The stronger red, the more not liked responses, and the stronger green, the less not liked responses.

	Urban		Pristine		Mature		Young	
	no.	%	no.	%	no.	%	no.	%
Airplane noise	13	20	42	64	19	29	28	42
Traffic noise	31	47	3	5	4	6	0	0
Characteristics of commercial forest	0	0	0	0	2	3	21	32
Dull	2	3	2	3	2	3	17	26
Dead branches	0	0	0	0	0	0	13	20
Other people	12	18	1	2	2	3	1	2
Thinning waste	0	0	0	0	0	0	11	17
Paths	9	14	0	0	0	0	0	0
Dead or decaying wood	0	0	4	6	0	0	0	0
Gloomy spruces	2	3	1	2	3	5	0	0
Dark	1	2	0	0	2	3	3	5
Mosquitos	0	0	1	2	1	2	3	5
Thicket	0	0	0	0	0	0	3	5
Rubbish	1	2	2	3	1	2	0	0
Worn spots	2	3	0	0	0	0	0	0
Fear of downfall dead trees	0	0	2	3	0	0	0	0
Sadness	0	0	1	2	0	0	1	2
Familiarity	1	2	0	0	0	0	1	2
No answer	9	14	5	8	18	27	4	6

**Table 6:** Final model (step three) for the multiple regression analyses for variables predicting overall perceived restorativeness (PRS-score) in four different forests.

	Urban				Pristine				Mature				Young			
Step 3	B	Std. Error	β	*p*	B	Std. Error	β	*p*	B	Std. Error	β	*p*	B	Std. Error	β	*p*
(Constant)	0.73	1.11		0.517	0.69	0.68		0.314	0.13	0.89		0.882	1.91	0.98		0.057
Temperature, °C	0.01	0.02	0.06	0.582	−0.01	0.02	−0.03	0.749	0.02	0.01	0.20	0.041	−0.01	0.02	−0.03	0.720
Gender: 0 = Men, 1 = Women	−0.06	0.20	−0.03	0.775	−0.05	0.13	−0.03	0.729	−0.23	0.15	−0.15	0.121	0.12	0.19	0.05	0.527
Age	0.01	0.01	0.07	0.536	0.00	0.01	0.02	0.755	0.01	0.01	0.11	0.216	−0.01	0.01	−0.07	0.387
Educational level: Other = 0, Uni = 1	−0.10	0.23	−0.05	0.686	−0.32	0.14	−0.17	0.031	0.13	0.16	0.08	0.426	0.01	0.21	0.00	0.968
Childhood environment: Countryside = 0, City = 1	0.07	0.25	0.03	0.790	−0.20	0.17	−0.09	0.261	−0.20	0.19	−0.10	0.293	−0.13	0.24	−0.05	0.593
Nature Relatedness, NR-6	0.26	0.18	0.18	0.150	0.30	0.12	0.23	0.013	0.33	0.13	0.27	0.014	0.12	0.16	0.07	0.461
Familiarity of outdooring in forest	−0.09	0.13	−0.08	0.523	0.02	0.09	0.02	0.836	0.06	0.10	0.06	0.564	−0.02	0.12	−0.01	0.869
Work related to nature: No = 0, Yes = 1	0.06	0.22	0.03	0.799	−0.15	0.15	−0.08	0.349	0.05	0.17	0.03	0.778	0.09	0.23	0.04	0.697
Beautiful	0.31	0.12	0.37	0.016	0.37	0.08	0.52	0.001	0.33	0.10	0.42	0.002	0.34	0.09	0.54	0.001
Safe	0.16	0.11	0.18	0.135	0.03	0.07	0.04	0.641	0.07	0.10	0.08	0.493	0.13	0.07	0.19	0.085
Bright	0.02	0.09	0.03	0.795	0.00	0.06	0.01	0.939	−0.02	0.06	−0.04	0.729	−0.12	0.08	−0.17	0.175
Managed	−0.04	0.06	−0.08	0.499	0.14	0.05	0.21	0.012	−0.01	0.05	−0.03	0.780	0.01	0.06	0.01	0.901
Rich in biodiversity	0.13	0.08	0.20	0.106	0.19	0.08	0.24	0.025	0.18	0.07	0.29	0.013	0.23	0.07	0.38	0.002

Note: B = regression coefficient, β = standardized regression coefficient. R^2^ = coefficient of determinations. 95% Confidence Intervals and t-values are described in Table S5, in the Supplementary Materials.

## Data Availability

Data sharing not applicable due to the ethical issues agreed with the participants.

## Supplementary Materials

The following are available online at https://www.mdpi.com/1660-4601/18/2/422/s1, Table S1: Background information of the participants (percentages), Table S2: Nature Relatedness Scale (NR-6) items, Table S3: Perceived Restorativeness Scale (PRS) components and items, Table S4: Scale statistics of environmental variables in four forests during the experiment, Table S5: The whole model for the multiple regression analyses for variables predicting overall perceived estorativeness (PRS-score) in four different forests, Table S6: Correlations between PRS components, PRS-score, semantic differential adjective pair, sound focus other than nature sound and temperature in Urban forest after the experiment, Table S7: Correlations between PRS components, PRS-score, semantic differential adjective pair, sound focus other than nature sound and temperature in Pristine forest after the experiment, Table S8: Correlations between PRS components, PRS-score, semantic differential adjective pair, sound focus other than nature sound and temperature in Mature forest after the experiment, Table S9: Correlations between PRS components, PRS-score, semantic differential adjective pair, sound focus other than nature sound and temperature in Young forest after the experiment, Table S10: Correlations or nonparametric test values of the null hypothesis between the PRS-score and temperature, individual variables and five adjective-pairs that were chosen into the multiple regression model in Urban forest, Table S11: Correlations or nonparametric test values of the null hypothesis between the PRS-score and temperature, individual variables and five adjective-pairs that were chosen in to the multiple regression model in Pristine forest, Table S12: Correlations or nonparametric test values of the null hypothesis between the PRS-score and temperature, individual variables and five adjective-pairs that were chosen into the multiple regression model in Mature forest, Table S13: Correlations or nonparametric test values of the null hypothesis between the PRS-score and temperature, individual variables and five adjective-pairs that were chosen in to the multiple regression model in Young forest.
