# Supplementary material for: The Perceived Restorativeness of Differently Managed Forests and Its Association with Forest Qualities and Individual Variables: A Field Experiment
Source: Int J Environ Res Public Health. 2021 Jan 7;18(2):422. doi: 10.3390/ijerph18020422 (PMC7825791; doi:10.3390/ijerph18020422)
Supplement: Supplementary file 1 [file ijerph-18-00422-s001.pdf]

Supplementary

Table S1. Background information of the participants (percentages).

Background information	%
Gender	
Women	59
Men	41
Age, years	
26-35	32
36-45	26
46-55	27
56-65	15
Childhood dwelling area	
Urban centre	15
City suburb	34
Municipality centre	12
Municipality suburb	21
Countryside or sparsely populated area	18
Education	
Academic degree (university of applied sciences, bachelor, master, PhD)	74
Short-cycle tertiary education	12
High school	9
Vocational/basic level	5
Job related to nature	23
Education related to nature	21
Forest owners	9

Table S2. Nature Relatedness Scale (NR-6) items.

Items
My ideal vacation spot would be a remote, wilderness area.
I always think about how my actions affect the environment.
My connection to nature and the environment is a part of my spirituality.
I take notice of wildlife wherever I am.
My relationship to nature is an important part of who I am.
I feel very connected to all living things and the earth.

Table S3. Perceived Restorativeness Scale (PRS) components and items.

Components	Items
Being away	It is a place to get away from it all
Being away	Spending time here gives me a break from my day-to-day routine
Fascination	My attention is drawn to many interesting things
Fascination	The setting is fascinating
Fascination	There is much to explore and discover here
Fascination	I want to get to know this place better
Fascination	I want to spend more time looking at the surroundings
Compatibility	Being here suits my personality
Compatibility	I can find ways to enjoy myself here
Compatibility	I have a sense that I belong here
Compatibility	I have a sense of oneness with this setting
Compatibility	I can do things I like here
Incoherence (Extent)	There is too much going on
Incoherence (Extent)	It is a confusing place
Incoherence (Extent)	There is a great deal of distraction
Incoherence (Extent)	It is a chaotic place

Note: The reliability measured with Cronbach´s α ranged for the PRS being away component from poor to acceptable, and from good to excellent for the PRS fascination and PRS compatibility components. However, for the PRS incoherence component, the Cronbach´s α ranged from an unacceptable to an acceptable level. In Finnish, the question 'It is a confusing place' can be interpreted as either meaning a positive or negative outcome, so the weak reliability scores were tested as they might be because of this question by

excluding it from the incoherence component. The reliability increased to an acceptable level in all four forests with Cronbach´s alphas: Urban=.87, Pristine=.74, Mature=.78, and Young=.82. However, this question was not removed from the PRS incoherence component as it had been validated in earlier studies and could not be removed [24].

Table S4. Scale statistics of environmental variables in four forests during the experiment.

**Forest site**	**Urban**			**Pristine**			**Mature**			**Young**		
Measures	Mean	SD	Cron α	Mean	SD	Cron α	Mean	SD	Cron α	Mean	SD	Cron α
Sound focus other than nature	3.48	1.5	-	4.14	1.63	-	3.11	1.40	-	3.95	1.48	-
Temperature, °C	14.8	4.4	-	15.8	4.2	-	15.9.	5.8	-	15.3	4.8	-
Noise, dBA	55.1	2.9	-	50.7	4.9	-	48.2	3.3	-	50.0	3.3	-

Results from the surrounding environmental variables

Other than nature sounds (sound focus other than nature) captured one's attention significantly more in the old-growth ($p < .01$) and young commercial forests ($p < .01$) than in the mature commercial forest, and more in the old-growth forest ($p \leq .01$) than in the urban recreation forest. The average noise level (dBA) was significantly higher in the urban recreation forest compared to the other three forests ($p < .01$), but there was no correlation between the average noise (dBA) and components of the PRS in different forests on the Spearman correlation.

There were no significant differences on temperatures between the forests, but there was a correlation between some components of the PRS and the average temperature on the Pearson correlation in a young commercial forest and a mature commercial forest. Please see Table D above for sound focus other than nature, noise level and temperature.

Table S5. The whole model for the multiple regression analyses for variables predicting overall perceived restorativeness (PRS-score) in four different forests.

		Urban							Pristine							Mature							Young						
		B	Std. Error	Std. Beta	t	p	95% CI		B	Std. Error	Std. Beta	t	p	95% CI		B	Std. Error	Std. Beta	t	p	95% CI		B	Std. Error	Std. Beta	t	p	95% CI	
Step 1	(Constant)	4.85	0.69		7.04	0.00	3.47	6.22	6.11	0.61		9.99	0.00	4.88	7.33	5.37	0.55		9.78	0.00	4.27	6.46	5.78	0.81		7.15	0.00	4.16	7.40
	Temperature, °C	0.02	0.02	0.13	1.00	0.32	-0.02	0.07	0.00	0.03	-0.01	-0.11	0.91	-0.06	0.05	0.03	0.02	0.21	1.69	0.10	0.00	0.06	**-0.07**	**0.03**	**-0.29**	**-2.38**	**0.02**	**-0.13**	**-0.01**
	Gender 0=men, 1=women	0.07	0.23	0.04	0.30	0.77	-0.39	0.52	0.27	0.21	0.16	1.32	0.19	-0.14	0.68	-0.04	0.20	-0.02	-0.19	0.85	-0.43	0.36	0.24	0.28	0.11	0.85	0.40	-0.32	0.80
	Age	0.01	0.01	0.09	0.72	0.47	-0.01	0.03	0.00	0.01	-0.03	-0.26	0.79	-0.02	0.02	0.01	0.01	0.09	0.70	0.49	-0.01	0.02	0.00	0.01	-0.04	-0.29	0.77	-0.03	0.02
	Educational level: Other=0, Uni=1	**-0.61**	**0.25**	**-0.31**	**-2.46**	**0.02**	**-1.11**	**-0.11**	**-0.60**	**0.23**	**-0.32**	**-2.67**	**0.01**	**-1.05**	**-0.15**	-0.29	0.21	-0.17	-1.36	0.18	-0.71	0.14	-0.49	0.30	-0.20	-1.61	0.11	-1.10	0.12
	Childhood residency: Countryside=0, City=1	0.14	0.28	0.06	0.49	0.63	-0.43	0.71	-0.25	0.27	-0.12	-0.93	0.36	-0.78	0.29	-0.15	0.24	-0.08	-0.64	0.53	-0.64	0.33	0.15	0.35	0.05	0.43	0.67	-0.55	0.85

Note. Urban: R2 adj. = .04, Pristine: R2 adj. = .07, Mature: R2 adj. = .03, Young: R2 adj. = .06.

		B	Std. Error	Std. Beta	t	p	95% CI		B	Std. Error	Std. Beta	t	p	95% CI		B	Std. Error	Std. Beta	t	p	95% CI		B	Std. Error	Std. Beta	t	p	95% CI	
Step 2	(Constant)	3.47	1.07		3.23	0.00	1.32	5.63	3.58	0.88		4.06	0.00	1.82	5.34	3.67	0.83		4.43	0.00	2.01	5.32	5.80	1.22		4.76	0.00	3.36	8.24
	Temperature, °C	0.02	0.02	0.13	1.06	0.29	-0.02	0.07	0.00	0.03	0.00	0.01	1.00	-0.05	0.05	0.02	0.01	0.18	1.54	0.13	-0.01	0.05	**-0.06**	**0.03**	**-0.24**	**-2.02**	**0.05**	**-0.12**	**0.00**
	Gender 0=men, 1=women	0.01	0.23	0.01	0.05	0.96	-0.46	0.48	0.22	0.19	0.13	1.16	0.25	-0.16	0.60	-0.11	0.19	-0.07	-0.58	0.56	-0.50	0.27	0.17	0.28	0.08	0.60	0.55	-0.39	0.73
	Age	0.01	0.01	0.08	0.66	0.51	-0.01	0.03	0.00	0.01	-0.05	-0.48	0.64	-0.02	0.01	0.01	0.01	0.07	0.62	0.54	-0.01	0.02	0.00	0.01	-0.04	-0.31	0.76	-0.03	0.02
	Educational level: Other=0, Uni=1	**-0.51**	**0.25**	**-0.25**	**-2.01**	**0.05**	**-1.01**	**0.00**	-0.40	0.21	-0.22	-1.95	0.06	-0.81	0.01	-0.15	0.20	-0.09	-0.72	0.47	-0.56	0.26	-0.40	0.30	-0.16	-1.32	0.19	-1.00	0.20
	Childhood residency: Countryside=0, City=1	0.00	0.30	0.00	0.01	0.99	-0.60	0.60	-0.49	0.25	-0.23	-1.98	0.05	-0.99	0.01	-0.35	0.24	-0.18	-1.46	0.15	-0.84	0.13	-0.09	0.36	-0.03	-0.26	0.80	-0.81	0.62
	Nature Relatedness, NR6	0.38	0.20	0.26	1.87	0.07	-0.03	0.79	**0.62**	**0.17**	**0.46**	**3.69**	**0.00**	**0.28**	**0.96**	**0.53**	**0.16**	**0.43**	**3.21**	**0.00**	**0.20**	**0.86**	0.24	0.24	0.13	0.98	0.33	-0.25	0.72
	Familiarity of outdooring in forest	-0.01	0.15	-0.01	-0.08	0.94	-0.32	0.30	0.06	0.13	0.05	0.43	0.67	-0.20	0.32	-0.04	0.12	-0.04	-0.33	0.74	-0.29	0.21	-0.17	0.18	-0.13	-0.95	0.35	-0.54	0.19
	Work related to nature: No=0, Yes=1	0.07	0.26	0.03	0.27	0.78	-0.45	0.59	-0.26	0.22	-0.13	-1.18	0.24	-0.71	0.18	0.08	0.21	0.05	0.39	0.70	-0.34	0.50	**-0.78**	**0.31**	**-0.30**	**-2.52**	**0.01**	**-1.41**	**-0.16**

Note. Urban: R2 adj. = .06, Pristine: R2 adj. = .27*, Mature: R2 adj. = .15*, Young: R2 adj. = .14*.**

		B	Std. Error	Std. Beta	t	p	95% CI		B	Std. Error	Std. Beta	t	p	95% CI		B	Std. Error	Std. Beta	t	p	95% CI		B	Std. Error	Std. Beta	t	p	95% CI	
Step 3	(Constant)	0.73	1.11		0.65	0.52	-1.51	2.96	0.69	0.68		1.02	0.31	-0.67	2.05	0.13	0.89		0.15	0.88	-1.64	1.91	1.91	0.98		1.95	0.06	-0.06	3.87
	Temperature, °C	0.01	0.02	0.06	0.55	0.58	-0.03	0.05	-0.01	0.02	-0.03	-0.32	0.75	-0.05	0.03	**0.02**	**0.01**	**0.20**	**2.09**	**0.04**	**0.00**	**0.05**	-0.01	0.02	-0.03	-0.36	0.72	-0.05	0.03
	Gender 0=men, 1=women	-0.06	0.20	-0.03	-0.29	0.77	-0.47	0.35	-0.05	0.13	-0.03	-0.35	0.73	-0.31	0.22	-0.23	0.15	-0.15	-1.58	0.12	-0.53	0.06	0.12	0.19	0.05	0.64	0.53	-0.26	0.50
	Age	0.01	0.01	0.07	0.62	0.54	-0.01	0.02	0.00	0.01	0.02	0.31	0.75	-0.01	0.01	0.01	0.01	0.11	1.25	0.22	0.00	0.02	-0.01	0.01	-0.07	-0.87	0.39	-0.02	0.01
	Educational level: Other=0, Uni=1	-0.10	0.23	-0.05	-0.41	0.69	-0.56	0.37	**-0.32**	**0.14**	**-0.17**	**-2.22**	**0.03**	**-0.61**	**-0.03**	0.13	0.16	0.08	0.80	0.43	-0.20	0.46	0.01	0.21	0.00	0.04	0.97	-0.41	0.43
	Childhood residency: Countryside=0, City=1	0.07	0.25	0.03	0.27	0.79	-0.44	0.58	-0.20	0.17	-0.09	-1.14	0.26	-0.54	0.15	-0.20	0.19	-0.10	-1.06	0.29	-0.57	0.18	-0.13	0.24	-0.05	-0.54	0.59	-0.62	0.36
	Nature Relatedness, NR6	0.26	0.18	0.18	1.46	0.15	-0.10	0.62	**0.30**	**0.12**	**0.23**	**2.57**	**0.01**	**0.07**	**0.54**	**0.33**	**0.13**	**0.27**	**2.55**	**0.01**	**0.07**	**0.59**	0.12	0.16	0.07	0.74	0.46	-0.20	0.44
	Familiarity of outdooring in forest	-0.09	0.13	-0.08	-0.64	0.52	-0.35	0.18	0.02	0.09	0.02	0.21	0.84	-0.16	0.19	0.06	0.10	0.06	0.58	0.56	-0.14	0.26	-0.02	0.12	-0.01	-0.17	0.87	-0.27	0.23
	Work related to nature: No=0, Yes=1	0.06	0.22	0.03	0.26	0.80	-0.39	0.51	-0.15	0.15	-0.08	-0.94	0.35	-0.46	0.16	0.05	0.17	0.03	0.28	0.78	-0.29	0.38	0.09	0.23	0.04	0.39	0.70	-0.38	0.56
	Beautiful	**0.31**	**0.12**	**0.37**	**2.49**	**0.02**	**0.06**	**0.56**	**0.37**	**0.08**	**0.52**	**4.67**	**0.00**	**0.21**	**0.53**	**0.33**	**0.10**	**0.42**	**3.30**	**0.00**	**0.13**	**0.53**	**0.34**	**0.09**	**0.54**	**3.90**	**0.00**	**0.17**	**0.52**
	Safe	0.16	0.11	0.18	1.52	0.13	-0.05	0.37	0.03	0.07	0.04	0.47	0.64	-0.11	0.18	0.07	0.10	0.08	0.69	0.49	-0.14	0.28	0.13	0.07	0.19	1.75	0.09	-0.02	0.27
	Bright	0.02	0.09	0.03	0.26	0.80	-0.16	0.20	0.00	0.06	0.01	0.08	0.94	-0.12	0.13	-0.02	0.06	-0.04	-0.35	0.73	-0.15	0.10	-0.12	0.08	-0.17	-1.37	0.18	-0.28	0.05
	Managed	-0.04	0.06	-0.08	-0.68	0.50	-0.17	0.08	**0.14**	**0.05**	**0.21**	**2.60**	**0.01**	**0.03**	**0.25**	-0.01	0.05	-0.03	-0.28	0.78	-0.11	0.08	0.01	0.06	0.01	0.13	0.90	-0.11	0.13
	Rich in biodiversity	0.13	0.08	0.20	1.64	0.11	-0.03	0.29	**0.19**	**0.08**	**0.24**	**2.31**	**0.02**	**0.03**	**0.36**	**0.18**	**0.07**	**0.29**	**2.57**	**0.01**	**0.04**	**0.33**	**0.23**	**0.07**	**0.38**	**3.29**	**0.00**	**0.09**	**0.38**

Note. Urban: R2 adj. = .33*, Pristine: R2 adj. = .68***, Mature: R2 adj. = .52***, Young: R2 adj. = .63***.**

Note: ***Step is significant at a level of $p < .001$, **at a level of $p < .01$ level and *at a level of $p < .05$. B = regression coefficient, standardized beta = β. R^2=coefficient of determinations. CI = Confidence Intervals.

Detailed description of steps 1 and 2, (the step 3 can be seen in the article)

In step 1, the higher educational level (Uni) was negatively associated to the perceived restorativeness in urban recreation forest (β=-.31, p=0.017) and in old-growth forest (β=-.32, p=0.010). Also the higher temperature was negatively associated to the perceived restorativeness in young commercial forest (β=-.29, p=0.020) but not in other three forests.

When adding the variables in the step 2, that describes the participant´s relationship with nature (see Table A), the model become significant in old-growth forest (p=0.001), mature commercial forest (*p*=0.012) and in young commercial forest (p=0.050) with corresponding coefficient of determinations of 27%, 15% and 14% of the variation of perceived restorativeness. The Nature Relatedness had positive association with the perceived restorativeness in old-growth forest (β=.46, p=0.001) and in mature commercial forest (β=.43, p=0.002) while the association with the educational level in step 1 disappeared in the old-growth forest. The childhood spent in the city was close to significant association with less effective restorativeness in the old-growth forest than the childhood spent in the countryside (β=-.23, p=0,053). The work related to nature was negatively associated to the perceived restorativeness in the young commercial forest (β=-.30, p=0.015), where also the negative connection with higher temperature remained (β=.-24, p=0.048). The negative connection remained in the urban recreation forest although it was now smaller (β=-.25, p=0.049).

Multicollinearity can be detected from tolerance and VIF-values. The tolerance-value should not be below 0.2, and if it is less than 0.4, then there is some concern, whereas the VIF-value should not be above 5.0. In the final multiple regression model (step 3), there was no multicollinearity detected among individual variables with the lowest tolerance value of 0.630 and the highest VIF value of 1.588 (in the case of Nature Relatedness Scale in the old-growth forest). The lowest tolerance value of 0.298, and the VIF value of 3.357 among the forest qualities was detected for the adjective-pair in the case of beautiful-ugly in the young commercial forest, indicating acceptable multicollinearity with some concerns, and therefore the model is approximate. Each model obtained acceptable results indicating no autocorrelation from the Durbin Watson test with values ranging between 1.698-2.630 (acceptable values 0-4).

Table S6. Correlations between PRS components, PRS-score, semantic differential adjective pair, sound focus other than nature sound and temperature in Urban forest after the experiment.

Urban	PRS fascination	PRS compatibility	PRS incoherence/extent	PRS-score	Pleasant-Unpleasant	Beautiful-Ugly	Safe-Scary	Restorative-Stressful	Rich-Poor in biodiversity	Natural-Artificial	Interesting-Dull	Calm-Restless	Harmonious-Chaotic	Bright-Dark	Cheerful-Sad	Managed-Unmanaged	Sound focus other than nature	Temperature, °C
PRS beingaway	.694**	.740**	-.487**	.847**	.571**	.423**	.317**	.684**	.320**	.565**	.506**	.540**	.430**	0.052	.361**	-0.100	-.283*	0.130
PRS fascination		.798**	-.275*	.861**	.608**	.471**	.408**	.695**	.321**	.439**	.581**	.544**	.505**	0.173	.459**	0.066	-.337**	0.092
PRS compatibility			-.405**	.918**	.639**	.536**	.392**	.692**	.374**	.511**	.597**	.593**	.507**	0.192	.331**	0.029	-.368**	0.116
PRS incoherence / extent				-.641**	-.600**	-.456**	-.264*	-.549**	-.270*	-.393**	-.480**	-.709**	-.555**	-0.121	-.289*	0.021	.610**	0.019
PRS-score					.751**	.600**	.433**	.801**	.402**	.579**	.683**	.739**	.632**	0.180	.447**	0.011	-.499**	0.074
Pleasant-Unpleasant						.790**	.410**	.702**	.473**	.605**	.720**	.752**	.700**	0.185	.498**	0.042	-.471**	0.006
Beautiful-Ugly							.458**	.686**	.452**	.535**	.686**	.614**	.721**	.398**	.614**	0.211	-.396**	0.029
Safe-Scary								.403**	0.104	.392**	.370**	.384**	.440**	0.080	0.226	0.028	-0.087	0.042
Restorative-Stressful									.533**	.544**	.661**	.776**	.585**	0.192	.476**	0.057	-.508**	-0.097
Rich-Poor in biodiversity										.578**	.469**	.485**	.316**	0.125	.500**	-0.058	-.260*	0.089
Natural-Artificial											.547**	.554**	.455**	0.102	.468**	-0.179	-.267*	0.070
Interesting-Dull												.735**	.639**	.256*	.627**	0.100	-.441**	0.001
Calm-Restless													.710**	0.214	.497**	0.054	-.657**	-0.074
Harmonious-Chaotic														.367**	.544**	.263*	-.446**	0.002
Bright-Dark															.609**	.460**	0.089	0.081
Cheerful-Sad																.269*	-0.132	0.014
Managed-Unmanaged																	-0.096	-0.035
Sound focus other than nature																		-0.042

Note. **. Correlation is significant at p < .01 level (2-tailed). *. Correlation is significant at a level of p < .05 (2-tailed).

Table S7. Correlations between PRS components, PRS-score, semantic differential adjective pair, sound focus other than nature sound and temperature in Pristine forest after the experiment.

Pristine	PRS fascination	PRS compatibility	PRS incoherence/extent	PRS-score	Pleasant-Unpleasant	Beautiful-Ugly	Safe-Scary	Restorative-Stressful	Rich-Poor in biodiversity	Natural-Artificial	Interesting-Dull	Calm-Restless	Harmonious-Chaotic	Bright-Dark	Cheerful-Sad	Managed-Unmanaged	Sound focus other than nature	Temperature, °C
PRS beingaway	.757**	.798**	-.278*	.830**	.564**	.601**	.353**	.595**	.342**	.507**	.568**	.428**	.544**	.374**	.368**	-0.056	-0.17	0.00
PRS fascination		.807**	-0.207	.858**	.694**	.696**	.425**	.636**	.479**	.507**	.679**	.416**	.521**	.368**	.456**	-0.019	-0.06	-0.06
PRS compatibility			-.358**	.925**	.680**	.702**	.387**	.732**	.514**	.486**	.614**	.530**	.661**	.492**	.487**	0.0126	-0.13	-0.04
PRS incoherence / extent				-.598**	-.496**	-.513**	-.356**	-.396**	-0.23	-.280*	-0.2166	-.659**	-.509**	-0.185	-.369**	-0.168	.307*	-0.05
PRS-score					.768**	.790**	.478**	.741**	.503**	.544**	.643**	.647**	.703**	.445**	.536**	0.0473	-0.21	-0.02
Pleasant-Unpleasant						.851**	.553**	.735**	.670**	.609**	.702**	.510**	.671**	.444**	.412**	-0.201	0.013	-0.02
Beautiful-Ugly							.534**	.752**	.580**	.531**	.679**	.557**	.627**	.453**	.482**	-0.112	-0.04	-0.06
Safe-Scary								.439**	.416**	.349**	.269*	.317**	.444**	.255*	0.2374	-0.104	.302*	0.18
Restorative-Stressful									.625**	.556**	.625**	.631**	.586**	.429**	.475**	-0.219	-0.08	-0.05
Rich-Poor in biodiversity										.469**	.646**	.258*	.475**	.309*	.382**	-.367**	0.15	0.01
Natural-Artificial											.567**	.403**	.372**	.346**	.457**	-.274*	-0.02	-0.08
Interesting-Dull												.402**	.463**	.287*	.355**	-.384**	-0.13	-0.23
Calm-Restless													.479**	.276*	.375**	-0.018	-.438**	-.262*
Harmonious-Chaotic														.472**	.414**	-0.116	-0.04	0.10
Bright-Dark															.561**	0.0291	0.041	.246*
Cheerful-Sad																0.0845	-0.1	0.10
Managed-Unmanaged																	-0.08	0.11
Sound focus other than nature																		.363**

Note. **. Correlation is significant at $p < .01$ level (2-tailed). *. Correlation is significant at a level of $p < .05$ (2-tailed).

Table S8. Correlations between PRS components, PRS-score, semantic differential adjective pair, sound focus other than nature sound and temperature in Mature forest after the experiment.

Mature	PRS fascination	PRS compatibility	PRS incoherence/extent	PRS-score	Pleasant-Unpleasant	Beautiful-Ugly	Safe-Scary	Restorative-Stressful	Rich-Poor in biodiversity	Natural-Artificial	Interesting-Dull	Calm-Restless	Harmonious-Chaotic	Bright-Dark	Cheerful-Sad	Managed-Unmanaged	Sound focus other than nature	Temperature, °C
PRS beingaway	.753**	.811**	-0.076	.823**	.520**	.338**	.293*	.469**	.552**	.518**	.453**	.427**	.397**	0.065	.304*	-.260*	-0.003	0.215
PRS fascination		.834**	-0.226	.926**	.676**	.670**	.334**	.640**	.605**	.585**	.717**	.489**	.572**	.385**	.489**	-0.179	-0.067	0.155
PRS compatibility			-.267*	.941**	.592**	.451**	.355**	.572**	.561**	.557**	.537**	.459**	.582**	0.181	.435**	-0.154	-0.008	0.174
PRS incoherence / extent				-.443**	-.352**	-.298*	-.447**	-.286*	-0.128	-0.233	-.243*	-.355**	-.259*	-0.135	-0.209	-0.023	.284*	-.266*
PRS-score					.685**	.583**	.433**	.638**	.597**	.605**	.644**	.540**	.595**	.274*	.475**	-0.175	-0.098	0.232
Pleasant-Unpleasant						.696**	.468**	.509**	.516**	.622**	.639**	.367**	.630**	.375**	.524**	-0.169	-0.108	0.116
Beautiful-Ugly							.444**	.633**	.554**	.668**	.830**	.364**	.693**	.523**	.571**	-0.167	-0.057	-0.009
Safe-Scary								.244*	.336**	.253*	.377**	.417**	.493**	.351**	.401**	-0.040	-0.005	0.227
Restorative-Stressful									.464**	.651**	.690**	.471**	.506**	.405**	.479**	-0.164	0.005	-0.082
Rich-Poor in biodiversity										.504**	.597**	.261*	.381**	.347**	.495**	-0.071	0.121	0.101
Natural-Artificial											.671**	.444**	.569**	.338**	.355**	-.395**	-0.009	-0.060
Interesting-Dull												.471**	.620**	.427**	.461**	-0.199	-0.096	0.034
Calm-Restless													.506**	.264*	.350**	-0.068	-0.240	0.234
Harmonious-Chaotic														.415**	.502**	-0.037	-0.031	-0.023
Bright-Dark															.640**	0.135	0.060	0.036
Cheerful-Sad																0.101	-0.058	0.091
Managed-Unmanaged																	0.109	0.050
Sound focus other than nature																		-0.167

Note. **. Correlation is significant at $p < .01$ level (2-tailed). *. Correlation is significant at a level of $p < .05$ (2-tailed).

Table S9. Correlations between PRS components, PRS-score, semantic differential adjective pair, sound focus other than nature sound and temperature in Young forest after the experiment.

Young	PRS fascination	PRS compatibility	PRS incoherence/extent	PRS-score	Pleasant-Unpleasant	Beautiful-Ugly	Safe-Scary	Restorative-Stressful	Rich-Poor in biodiversity	Natural-Artificial	Interesting-Dull	Calm-Restless	Harmonious-Chaotic	Bright-Dark	Cheerful-Sad	Managed-Unmanaged	Sound focus other than nature	Temperature, °C
PRS beingaway	.666**	.691**	-.309*	.736**	.578**	.492**	.349**	.541**	.521**	.451**	.598**	.496**	.505**	.267*	.360**	-0.141	-0.040	-0.168
PRS fascination		.867**	-.480**	.931**	.756**	.683**	.496**	.661**	.709**	.590**	.800**	.538**	.657**	.490**	.639**	-0.177	-0.160	-.318**
PRS compatibility			-.517**	.944**	.743**	.707**	.517**	.690**	.661**	.608**	.664**	.475**	.669**	.524**	.678**	-0.076	-0.109	-.278*
PRS incoherence / extent				-.691**	-.619**	-.675**	-.519**	-.573**	-.445**	-.555**	-.445**	-.616**	-.694**	-.422**	-.463**	-0.138	.370**	0.153
PRS-score					.816**	.778**	.573**	.742**	.712**	.667**	.758**	.619**	.760**	.536**	.675**	-0.079	-0.207	-.291*
Pleasant-Unpleasant						.805**	.670**	.755**	.700**	.755**	.738**	.655**	.812**	.554**	.754**	-0.081	-0.132	-.366**
Beautiful-Ugly							.594**	.761**	.672**	.714**	.741**	.571**	.762**	.656**	.732**	-0.034	-0.149	-.272*
Safe-Scary								.723**	.441**	.559**	.495**	.537**	.670**	.601**	.569**	0.064	-.295*	-.271*
Restorative-Stressful									.621**	.666**	.695**	.614**	.719**	.612**	.636**	-0.037	-.263*	-0.208
Rich-Poor in biodiversity										.717**	.781**	.534**	.638**	.608**	.705**	-0.176	-0.177	-.359**
Natural-Artificial											.635**	.503**	.653**	.527**	.663**	-0.203	-0.173	-.367**
Interesting-Dull												.612**	.702**	.588**	.653**	-.302*	-0.137	-.327**
Calm-Restless													.704**	.349**	.412**	-0.048	-.274*	-.340**
Harmonious-Chaotic														.562**	.697**	-0.022	-.271*	-.343**
Bright-Dark															.675**	0.041	-0.168	-.310*
Cheerful-Sad																0.009	-0.173	-.308*
Managed-Unmanaged																	-0.120	-0.001
Sound focus other than nature																		0.169

Note. **. Correlation is significant at $p < .01$ level (2-tailed). *. Correlation is significant at a level of $p < .05$ (2-tailed).

Table S10. Correlations or nonparametric test values of the null hypothesis between the PRS-score and temperature, individual variables and five adjective-pairs that were chosen into the multiple regression model in Urban forest.

	Pearson correlation									*p-values: Dymmy coded/Mann-Whitney U Test*			
Urban	Temperature, °C	Age	Nature Relatedness, NR-6	Familiarity of outdooring in forest	Beautiful-Ugly	Safe-Scary	Rich-Poor in biodiversity	Bright-Dark	Managed-Unmanaged	Gender	Educational level	Childhood environment	Work related to nature
PRS-score	0.074	0.116	.311*	0.094	.600**	.433**	.402**	0.180	0.011	.855	.011*	.425	.939
Temperature, °C		-0.164	-0.057	-0.080	0.029	0.042	0.089	0.081	-0.035	.092	.367	.880	.414
Age			0.071	0.006	0.014	0.124	0.090	-0.102	-0.009	.477	.192	.739	.951
Nature Relatedness, NR-6				.361**	.279*	0.237	-0.006	0.045	0.084	.819	.084	.117	.782
Familiarity of outdooring in forest					0.183	0.019	0.137	0.146	0.046	.407	.816	.395	.280
Beautiful-Ugly						.458**	.452**	.398**	0.211	.583	.006**	.944	.584
Safe-Scary							0.104	0.080	0.028	.079	.024*	.606	.930
Rich-Poor in biodiversity								0.125	-0.058	.829	.207	.552	.855
Bright-Dark									.460**	.456	.312	.662	.642
Managed-Unmanaged										.573	.446	.939	.091

Note. **. Correlation is significant at $p < .01$ level (2-tailed). *. Correlation is significant at a level of $p < .05$ (2-tailed).

Table S11. Correlations or nonparametric test values of the null hypothesis between the PRS-score and temperature, individual variables and five adjective-pairs that were chosen in to the multiple regression model in Pristine forest.

	Pearson correlation									*p-values: Dymmy coded/Mann-Whitney U Test*			
Pristine	Temperature, °C	Age	Nature Relatedness, NR-6	Familiarity of outdooring in forest	Beautiful-Ugly	Safe-Scary	Rich-Poor in biodiversity	Bright-Dark	Managed-Unmanaged	Gender	Educational level	Childhood environment	Work related to nature
PRS-score	-0.022	0.028	.469**	0.211	.790**	.478**	.503**	.445**	0.047	.070	.007**	.136	.657
Temperature, °C		0.223	0.137	-0.156	-0.064	0.182	0.005	.246*	0.110	.623	.959	.051	.035*
Age			0.071	0.006	-0.002	-0.143	-0.105	0.102	0.008	.477	.192	.739	.951
Nature Relatedness, NR-6				.361**	.317**	.335**	0.181	.301*	0.005	.819	.084	.117	.782
Familiarity of outdooring in forest					0.118	0.044	0.145	0.117	-0.024	.407	.816	.395	.280
Beautiful-Ugly						.534**	.580**	.453**	-0.112	.022*	.150	.067	.463
Safe-Scary							.416**	.255*	-0.104	.031*	.932	.778	.342
Rich-Poor in biodiversity								.309*	-.367**	.068	.319	.398	.241
Bright-Dark									0.029	.924	.059	.876	.583
Managed-Unmanaged										.564	.938	.873	.429

Note. **. Correlation is significant at $p < .01$ level (2-tailed). *. Correlation is significant at a level of $p < .05$ (2-tailed).

Table S12. Correlations or nonparametric test values of the null hypothesis between the PRS-score and temperature, individual variables and five adjective-pairs that were chosen into the multiple regression model in Mature forest.

	Pearson correlation									*p-values: Dymmy coded/Mann-Whitney U Test*			
Mature	Temperature, °C	Age	Nature Relatedness, NR-6	Familiarity of outdooring in forest	Beautiful-Ugly	Safe-Scary	Rich-Poor in biodiversity	Bright-Dark	Managed-Unmanaged	Gender	Educational level	Childhood environment	Work related to nature
PRS-score	0.232	0.122	.409**	0.151	.583**	.433**	.597**	.274*	-0.175	.583	.123	.665	.280
Temperature, °C		0.053	0.070	-0.029	-0.009	0.227	0.101	0.036	0.050	.234	.617	.726	.407
Age			0.071	0.006	0.004	0.060	-0.017	0.113	-0.163	.477	.192	.739	.951
Nature Relatedness, NR-6				.361**	0.097	0.166	.270*	-0.083	-0.124	.819	.084	.117	.782
Familiarity of outdooring in forest					-0.132	0.198	-0.025	-0.158	-0.086	.407	.816	.395	.280
Beautiful-Ugly						.444**	.554**	.523**	-0.167	.135	.036*	.416	.702
Safe-Scary							.336**	.351**	-0.040	.320	.310	.370	.379
Rich-Poor in biodiversity								.347**	-0.071	.229	.149	.800	.224
Bright-Dark									0.135	.410	.237	.645	.975
Managed-Unmanaged										.250	.027*	.839	.523

Note. **. Correlation is significant at $p < .01$ level (2-tailed). *. Correlation is significant at a level of $p < .05$ (2-tailed).

Table S13. Correlations or nonparametric test values of the null hypothesis between the PRS-score and temperature, individual variables and five adjective-pairs that were chosen in to the multiple regression model in Young forest.

	Pearson correlation									*p-values: Dymmy coded/Mann-Whitney U Test*			
Young	Temperature, °C	Age	Nature Relatedness, NR-6	Familiarity of outdooring in forest	Beautiful-Ugly	Safe-Scary	Rich-Poor in biodiversity	Bright-Dark	Managed-Unmanaged	Gender	Educational level	Childhood environment	Work related to nature
PRS-score	-.291*	0.008	0.075	-0.129	.778**	.573**	.712**	.536**	-0.079	.662	.047*	.733	.003**
Temperature, °C		-0.020	0.097	0.011	-.272*	-.271*	-.359**	-.310*	-0.001	.187	.587	.920	.125
Age			0.071	0.006	0.060	-0.131	0.153	-0.021	-0.213	.477	.192	.739	.951
Nature Relatedness, NR-6				.361**	0.05	-0.135	0.041	-0.067	0.011	.819	.084	.117	.782
Familiarity of outdooring in forest					-0.142	-0.144	-0.116	-0.043	0.131	.407	.816	.395	.280
Beautiful-Ugly						.594**	.672**	.656**	-0.034	.858	.043*	.458	.000**
Safe-Scary							.441**	.601**	0.064	.679	.492	.634	.055
Rich-Poor in biodiversity								.608**	-0.176	.581	.120	.906	.003**
Bright-Dark									0.041	.448	.887	.355	.044*
Managed-Unmanaged										.070	.122	0.087	.839

Note. **. Correlation is significant at p < .01 level (2-tailed). *. Correlation is significant at a level of p < .05 (2-tailed).
